# An innovative inspection of a cantilever beam exposed to principal parametric excitation

**DOI:** 10.1038/s41598-025-24839-2

**Published:** 2025-11-18

**Authors:** Galal M. Moatimid, T. S. Amer, Mona A. A. Mohamed

**Affiliations:** 1 Department of Mathematics, Faculty of Education, Ain Shams University, Roxy, Cairo, Egypt; 2 Department of Mathematics, Faculty of Science, Tanta University, Tanta, 31527 Egypt

**Keywords:** Nonlinear oscillations, Cantilever beam, Non-perturbative approach, He’s frequency formula, Mathieu–Duffing oscillator, Chaotic motion, Bifurcation, Engineering, Mathematics and computing, Physics

## Abstract

The inspection of a cantilever beam subjected to parametric stimulation is essential in engineering structures such as bridges, aircraft wings, and micro electromechanical systems (MEMS). It is demonstrated that nonlinearities restrict the rise in accessibility. The existing issue mitigates vibrations in a structure exposed to primary parametric stimulation. It utilizes knowledge to develop a simple nonlinear feedback law designed to reduce vibrations of the first mode of a cantilever beam. The fundamental methodology relies on the non-perturbative approach (NPA), which is grounded in He’s frequency formula (HFF). This approach simply converts a weakly nonlinear oscillator of a second nonlinear ordinary differential equation (ODE) into a linear one. Consequently, the goal is to depart from traditional perturbation methods and get unrestricted approximation solutions of small amplitude parametric components. Furthermore, the method is extended to find the best solutions for large amplitude nonlinear fluctuations of coupled system. The generated parametric equation shows good agreement with the original equation when validated using the Mathematica Software (MS). The stability behavior is investigated across several contexts. The current methodology is founded on explicit principles, is suitable, user-friendly, and yields remarkably high numerical precision. The present approach reduces the mathematical difficulty, making it beneficial of the mathematical execution of nonlinear parametric issues. It is found that the system is stable with the increase of both linear and nonlinear damping coefficients, while it becomes less stable as excitation force’s parameters increase. Furthermore, the Poincaré map, phase portrait, and bifurcation are analyzed, which collectively provide a comprehensive depiction of the system’s behavior at different phases.

## Introduction

Previously, parametric resonance was discovered^1^. Horizontal surface waves are shown to be produced by a liquid in a vertically oscillating vessel. This resonance is unique because it produces high-amplitude movements when driving frequency gets close to double one of system’s intrinsic frequencies. Mathieu oscillator was the first how offers a model that showed this pattern^2^. Analysis of this model shows that viscous damping does not limit the response amplitude, unlike systems subjected to a primary external stimulation. On the other hand, instability is delayed by linear damping^3^. Theoretical findings corresponded well with the experimental results that were beforehand described^4^. Parametrically stimulated multi-degree-of-freedom systems are examined for their intriguing dynamics. An analysis was conducted on the system’s response comprising quadratic involved oscillators with circumstance of precise auto-parametric 2:1 resonance, whereby beneath mode is motivated at double its natural frequency^5^. It was established the criteria under which the system experiences Hopf bifurcations. A comparable system with cubic nonlinearities was examined^6^. For the combined track-based vision measuring system, an improved calibration technique was reported^7^. The system data transformation and scale factor were used to create a calibration equation. Orange and lime juices and peel extracts were used as natural reducing and stabilizing agents in a new, environmentally friendly microwave-assisted approach of synthesis of selenium nanoparticles^8^. A wavelength-stabilized, quasi-common-path heterodyne grating interferometer was presented to get over this restriction^9^. A self-injection locking laser model with two channels and whispering gallery mode was constructed^10^. A microresonator was intended to produce certain resonant modes. A three-step multi-task phase unwrapping technique was suggested as a solution to this problem^11^. One popular phase demodulation technique is internal modulation-based phase-generated carrier demodulation technology. Nevertheless, nonlinear errors are introduced under high-velocity and Largrange modulation and this approach were only appropriate for low-speed and small-distance measurement situations^12^. A fibre microprobe interference-sensing model and precision phase-generated carrier were combined to provide a novel sensing technique that enables large-range displacement measurement in limited space scenarios^13^. A dynamic vibration absorber based on particle damping technology was presented to regulate the roll’s vertical vibration, while rolling from the energy transfer and dissipation perspective^14^. There have been reports of high tracking accuracy and generalisation by frequency-domain iterative tuning^15^. Through the simultaneous detection of contact pressure and relative humidity distribution information, a self-sensing sealing electronic gasket with multi-modal interdigitated electrode-based sensors in evaluating underground pipeline damage was introduced^16^. Seven predamaged specimens reinforced with bond steel plate and high-performance ferrocement laminate were examined to determine the impact of pregavage level and strengthening procedures on the seismic performance of reinforced concrete columns^17^. The bearing capacities and deformation were computed using elastic and plastic analysis^18^.

In industries like flying, automotive, and civil engineering, where safety and performance optimization depend on an understanding of how structures react to various circumstances, this is especially important. Beams are used extensively at numerous sizes as basic structural elements. Advanced materials and technologies at the micro and nanoscales, as well as large-scale constructions like bridge spans, satellite components, and airplane wings, depend on them. A wide range of structural components may be examined and shown as beams. Beams are essential in aerospace industry of structures that must endure complicated dynamic pressures, such as satellite components and airplane wings^19^. Analysis was done on complex transverse vibrations of axially moving beams under harmonic stimulation, which are crucial in many structural and industrial applications^20^. In order to improve design and performance, the study aimed to comprehend dynamic behavior and precisely determine frequency responses. Many mathematicians have so been interested in asymptotic analysis of a number of nonlinear ODEs. To illustrate weak nonlinear oscillators of ODEs, averaging approach and lowest factor method were applied^21^. Accurate asymptotic computations of low-intensity acoustic events are obtained using homotopy perturbation method (HPM). Nonlinear ODEs may now be solved more easily thanks to the growing popularity of several HPM-based methods^22^. Should the beginning assumption not align with the chosen technique of inquiry, the procedure would turn off course and not yield the intended results. In order to examine analytical approximations of magnetic spherical pendulums, HPM was employed^23^. Nevertheless seeming advancement, HPM had difficulties when using non-conservative oscillators. The nonlinear vibration theory’s analytical capacity of improved HPM was ensured. In several different categories, coupled damping nonlinear oscillators are the main emphasis. HFF’s Hamiltonian function has garnered a lot of interest due to its capacity to streamline the computational analysis of intricate nonlinear vibration systems. To illustrate the computation’s precision and simplicity, the cubic-quintic DO was utilized. To solve the cubic-quintic DO, a straightforward approach was suggested^24^. The technique provided a very effective and partly precise way to determine frequency of a nonlinear conservative oscillator. The improved HFF of nonlinear oscillators was presented and demonstrated^25^. HFF was used to develop a direct frequency formula of fractal systems, which was published^26^. A fantastic instrument of in-depth analysis of fractal vibration occurrences was produced by simple calculations and trustworthy outcomes. Accordingly, studying these fluids is highly interesting. The evolution of many dynamical systems, including a flat disturbed interface, was illustrated. Our theoretical study’s main goal was to use NPA to supplement our most recent results^27–45^.

The investigation of cantilever beams under primary parametric stimulation has substantial experimental and practical relevance in numerous engineering and scientific domains. These investigations experimentally contribute to comprehension of dynamic stability, resonance signs, and nonlinear behavior of cantilever beams under time-varying loads or boundary conditions. Researchers utilize laboratory configurations featuring electromechanical shakers, piezoelectric actuators, and electromagnetic excitation systems to replicate real-world parametric excitation scenarios, facilitating accurate measurement of vibrations, amplitude responses, and stability thresholds through laser Doppler barometers and accelerometers. These results corroborate theoretical models and facilitate advancement of sophisticated computational methods of forecasting beam behavior under varying situations. Cantilever beams subjected to parametric excitation are essential in aerospace, civil, and mechanical engineering applications. Aerospace structures, specifically wing and blade components, experience parametric excitation from aerodynamic forces, necessitating stability analysis to avert catastrophic failures. Cantilever bridges and high-rise buildings in civil engineering, exposed to variable loads like wind and seismic forces, must be engineered to endure parametric resonance, hence maintaining structural integrity and safety. Additionally, in energy harvesting, parametric excitation methods are employed in piezoelectric cantilever beams to improve energy conversion efficiency from ambient vibrations, resulting in the creation of self-powered sensors and wearable electronics. Moreover, the MEMS systems and nanoscale devices utilize parametric excitation of precise control in biomedical applications, encompassing resonance-based sensing of detection of biological molecules and mechanical filtering in micro-scale devices. The examination of parametric excitation in cantilever beams is crucial of vibration isolation and suppression methods, including adaptive damping in precision engineering instruments and robotic arms, where undesirable oscillations may undermine accuracy and performance. The experimental and practical examination of cantilever beams subjected to parametric excitation is essential in enhancing structural health monitoring, refining design techniques, and creating creative solutions across several engineering fields. The following points are highlighted concerning the current topic:

(i) The current nonlinear ODE is comparable to its corresponding linear counterpart.
(ii) These two ODEs are similar when employing the method of successive approximations.
(iii) Each conventional approach utilizes Taylor expansion to assist in the resolution of restorative forces. The existing NPA resolves this matter.
(iv) Contrary to conventional procedures, NPA facilitates evaluation of problem’s stability analysis.
(v) The revolutionary technique appears to be a straightforward, appropriate, and interesting tool. It can be employed to the examination of diverse nonlinear oscillators.
(vi) NPA’s adaptability in handling a range of nonlinear problems makes it useful in applied research, science, and technology.

To simplify the presentation of the paper, the document’s structure is organized as follows: The problem formulation is quantified in section “Construction of issue”. The technique is presented in detailed in section “NPA exploration”. The presented methodology is devoted in this section, besides the validation of the results, analysis of the time-dependent behavior of the results, and stability analysis. In section “Analysis of chaotic behavior” the chaotic behavior of the examined system through analyzing the bifurcation, phase portrait, and Poincaré map are analyzed. The final part corroborates the main findings outlined in section “Conclusion”.

## Construction of issue

In practical applications, analyzing cantilever beams under primary parametric stimulation necessitates comprehension of how periodic axial forces affect vibrational properties, potentially resulting in resonance-induced instabilities that undermine structural integrity. This entails delineating stability zones, recognizing resonance circumstances, and evaluating effects of damping, boundary conditions, and nonlinearity. Advanced methodologies, including time-series analysis, bifurcation theory, and machine learning-based predictive modelling, improves the capacity to identify and alleviate instability issues. Experimental validation via laser barometer, strain gauge measurements, and real-time feedback control enhances theoretical models, guaranteeing precise predictions of applications in aeronautical, mechanical, and civil engineering structures. As previously shown, the main equation of the oscillator may be written as^46^:

1 $$\ddot{x} + x + 2\mu_{1} \dot{x} + \mu_{2} \left| {\dot{x}} \right|\dot{x} + \alpha_{1} x^{3} + \alpha_{2} x^{2} \ddot{x} + \alpha_{3} x\dot{x}^{2} + G\dot{x}^{3} - xF\cos \sigma t = 0,$$

where $\mu_{1}$ is viscous damping factor, and $x$ is actual coordinates. It takes internal material damping and external energy dissipation into account. The parameter $\mu_{2}$ is air drag coefficient. It models aerodynamic damping effects on beam. The parameters $\alpha_{i} (i = 1,\,2,\,3)$ are constants of nonlinear terms $\sigma$ and $F$ are frequency and forcing amplitude, respectively. The parameter $\alpha_{1}$ represents nonlinear curvature effects due to large deflections. The parameters $\alpha_{2}$ and $\alpha_{3}$ represent nonlinear inertia effects, capturing how beam’s motion affects its dynamic response. The term $\alpha_{1} x^{3}$ is due to nonlinear coefficient, and terms $\alpha_{2} x^{2} \ddot{x}$ and $\alpha_{3} x\dot{x}^{2}$ are due to nonlinear inertia^47^. The coefficient $G$ denotes control gain linked to cubic velocity feedback law employed to mitigate vibrations in cantilever beam. It introduces a nonlinear damping effect that stabilizes the system by constraining the amplitude of parametric resonance. Elevated values of $G$ augment vibration suppression reduce oscillation amplitude and boost transient response. This control technique is highly effective in eradicating unwanted bifurcations and maintaining system stability under parametric excitation. These coefficients delineate influence of several physical phenomena such as damping, nonlinear toughness, and external forcing on dynamic performance of cantilever beam subjected to parametric stimulation. $F$ denotes the excitation amplitude, while $\sigma$ is the excitation frequency. The last term in Eq. (1) represents the Mathieu excitation term which makes this equation somewhat as Mathieu nonlinear damping equation.

Equation (1) represents a nonlinear damped-driven oscillator with cubic and quadratic nonlinearities in both displacement and velocity, making it suitable of modeling real-world mechanical systems with complex friction, restoring forces, and excitation. This model could be applied to many dynamical systems like:

1. The MEMS devices with nonlinear electrostatic forces.
2. Mechanical structures under dynamic loads (e.g., beams, bridges, or panels with geometric nonlinearities).
3. Vehicle suspension systems (semi-active/active dampers).
4. Biomechanical systems involving muscle dynamics or soft tissue.
5. Seismically excited structures with base-isolation or energy dissipation devices.

## NPA exploration

**Fig. 1:**
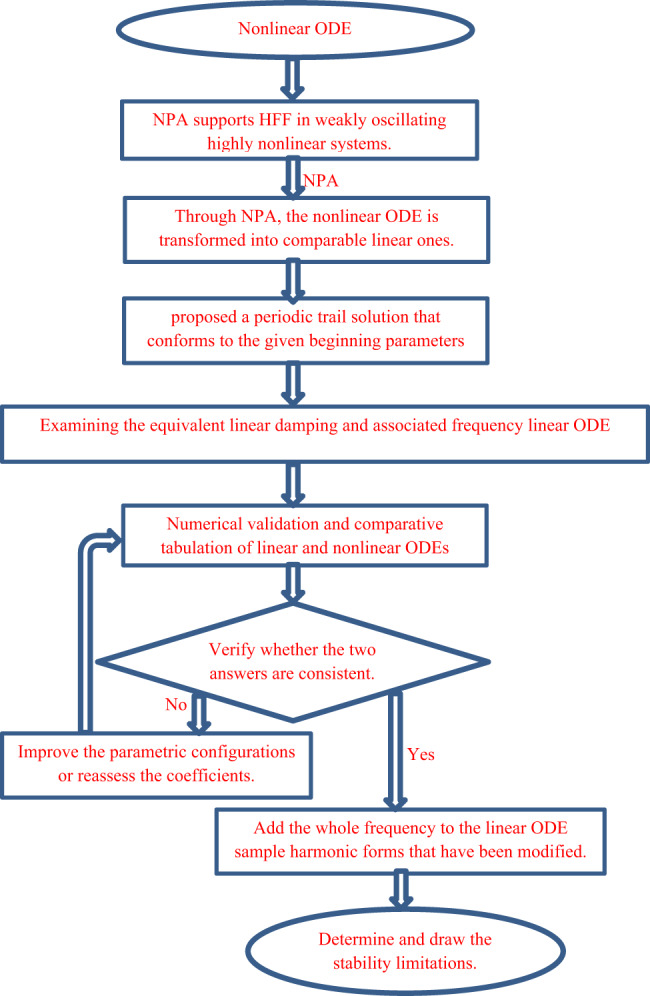
A flowchart clarifying the framework of NPA.

Concerning to NPA, there are many features unlike the distinctive classical perturbations methodology. For more convenience, a flowchart describing NPA is presented below in Fig. 1 . Many advantages are considered in this regard as:

1. The alternative equivalent linear ODE is created from a weakly nonlinear oscillator of ODE with periodic coefficients. Keep in mind that the linear ODE really contains all parameters in the nonlinear ODE.
2. As is generally known, all traditional perturbation techniques use Taylor expansion to reduce the complexity of comparable issues when restoring forces are present. This defectiveness is fixed by the current NPA.
3. In contrast to other traditional methods, the NPA enables the problem’s stability analysis to be examined.
4. The NPA appears to be an engaging, practical, and easy-to-use tool. It is applicable to the analysis of several types of nonlinear oscillators.

**Fig. 2:**
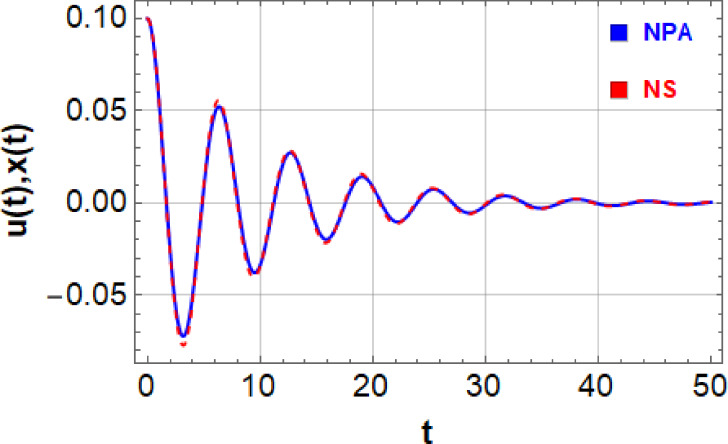
Shows the equivalence of the NSs of Eqs. (1) and (6).

Indeed, there are some limitations for the main aim of NPA. These restrictions may be summarized as follows:

(i) The methodology is valid only for weakly nonlinear oscillators of second-order ODE.
(ii) The initial conditions are fixed as given in all applications.
(iii) To attain a better accuracy, the initial amplitude must be less than unity.

Equation (1) could be seen as a nonlinear damped Duffing-Van der Pol-Mathieu oscillator (DVMO). As mentioned earlier, HFF is a crucial part of HPM architecture and was suggested by Prof. Ji-Huan He. The frequency of nonlinear oscillators is estimated using this formula, which combines HPM with an averaging technique^48–50^. For systems with significant nonlinearity, it provides a systematic expression that describes relationship concerning amplitude and fluctuation frequency. By integrating across a whole period, NPA makes use of system’s periodic properties. Integrating across one cycle of time that the oscillatory motion completes ensures that frequency estimation is in line with inherent dynamics of the system. The following benefits result from our long-term integration:

1. A thorough recording of nonlinear effects is made.
2. The entire activity or energy is preserved.
3. Higher-order nonlinearities are incorporated into the response function.

Because of amplitude dependency, the frequency of a nonlinear system deviates from linear case. This approach makes it possible to approximate this frequency without requiring series expansions, which makes it practical even when there are large nonlinearities. By efficiently balancing kinetic and potential energy, HFF’s integral form formula ensures that frequency estimation matches full energy distribution over a single cycle. Beyond theory of perturbation, NPA naturally incorporates important nonlinear elements, making it suitable of systems with huge amplitudes or hard/soft stiffness effects, unlike all conventional approaches that assume small modifications around a linearized system. Therefore, NPA, which is based on HFF and integral evaluation over A, provides a thorough way to study extremely nonlinear oscillators without being constrained by perturbation theory.

Concerning Eq. (1), the proposed initial conditions (ICs) are often expressed as:

2 $$x(0) = A,\,\,{\text{and}}\,\,\dot{x}(0) = 0.$$

Equation (1) could be re-formulated as:

3 $$\ddot{x} + f_{1} (x,\dot{x}) + f_{2} (x,\dot{x}) - xF\cos \sigma \,t = 0,$$

where $f_{1} (x,\dot{x})$ describes the odd damping terms and $f_{2} (x,\dot{x})$ signifies the secular odd terms:

where

4 $$\left. \begin{gathered} f_{1} (x,\dot{x}) = 2\mu_{1} \dot{x} + \mu_{2} \left| {\dot{x}} \right|\dot{x} + G\dot{x}^{3} \hfill \\ f_{2} (x,\dot{x}) = x + \alpha_{1} x^{3} + \alpha_{2} x^{2} \ddot{x} + \alpha_{3} x\dot{x}^{2} \hfill \\ \end{gathered} \right\}$$

Guessing a test solution of the DVMO, as given by Eq. (1) by:

5 $$\tilde{u} = A\cos \Omega t$$

where $\Omega$ is defined as the whole frequency to be estimated after.

Equation (3) may be rewritten, as^27–45^, concerning a new function $u$ as:

6 $$\ddot{u} + \tilde{\mu }\,\dot{u} + \left( {\tilde{\omega }^{2} - F\cos (\sigma \,t)} \right)u = 0$$

where $\tilde{\mu }$ and $\tilde{\omega }^{2}$ are the corresponding damping coefficient and corresponding natural frequency, individually. Equation (6) is the damped Mathieu equation.

It is noted that although Eq. (6) is a linear ODE, it has not an accurate solution due to existence of variable factor $F\cos (\sigma \,t)$. The unnamed coefficients performing in Eq. (6) are evaluated utilizing the integrals in light of the NPA^35,37^:

7 $$\tilde{\mu } = \int\limits_{0}^{2\pi /\Omega } {\dot{\tilde{u}}f_{1} (\tilde{u},\,\dot{\tilde{u}})} dt/\int\limits_{0}^{2\pi /\Omega } {\dot{\tilde{u}}^{2} } dt = 2\mu_{1} + \frac{{A^{2} }}{4}(3G + \mu_{2} )\Omega^{2},$$

and

8 $$\tilde{\omega }^{2} = \int\limits_{0}^{2\pi /\Omega } {\tilde{u}f_{2} (\tilde{u},\dot{\tilde{u}})\,dt/\int\limits_{0}^{2\pi /\Omega } {\tilde{u}^{2} dt} } = 1 + \frac{1}{4}A^{2} (3\alpha_{1} + (\alpha_{3} - 3\alpha_{2} ))\Omega^{2},$$

Note that the quadratic term $\mu_{2} \left| {\dot{x}} \right|\dot{x}$ is integrated with respect to $\dot{x}$ inside the integration in Eq. (7) to catch its coefficient in the equivalent damping $\tilde{\mu }$. The normal formula approach can be used to represent Eq. (6) in its standard form. Consequently, it is reasonable to suggest the following modification:

9 $$u = e^{{ - (\tilde{\mu }/2\,)t}} g(t).$$

Now, $g(t)$ is the required function to be evaluated.

Inserting Eq. (9) in Eq. (6), the ODE that governs the unidentified function $g(t)$ can be realized as:

10 $$\ddot{g} + \left( {\Omega^{2} - F\cos \sigma \,t} \right)g = 0.$$

which is the conventional Mathieu oscillator with a natural frequency $\Omega$, expressed as.

11 $$\begin{aligned} \Omega ^{2} & = \tilde{\omega }^{2} - \frac{1}{4}\tilde{\mu }^{2} \\ & = \frac{1}{{9A^{4} G^{2} + A^{4} \mu _{2}^{2} (6G + 1)}} \\ & \;\;\left( {\begin{array}{*{20}l} { - 32 - 24A^{2} \alpha _{2} + 8A^{2} \alpha _{3} - 24A^{2} G\mu _{1} - 8A^{2} \mu _{1} \mu _{2} + } \hfill \\ {\frac{1}{2}\sqrt[{}]{{(64 + 48A^{2} \alpha _{2} - 16A^{2} \alpha _{3} + 48A^{2} G\mu _{1} + 16A^{2} \mu _{1} \mu _{2} )^{2} - 4( - 64 - 48A^{2} \alpha _{1} + 64\mu _{1}^{2} )(9A^{4} G^{2} + A^{4} \mu _{2}^{2} (6G + 1))}}} \hfill \\ \end{array} } \right). \\ \end{aligned}$$

Once again, the periodic coefficients make it impossible to find an accurate solution to Eq. (10) even if it is a linear ODE. Furthermore, it is difficult to fulfill the stability criteria. As mentioned before, NPA will be adjusted to accommodate these challenges^27–45^. The current objective is to convert Eq. (10) into a different equivalent equation with constant coefficients in the way that follows:

12 $$\ddot{h} + \Sigma^{2} (\Omega )h = 0.$$

The linear Mathieu equation is provided by Eq. (6). In fact, it lacks an accurate solution because of the presence of the time-dependent factor. Once again, the multiple-time scales method was widely employed in classical perturbation techniques. In contrast, Eq. (12), which is precisely a simple harmonic equation, is produced by evolution of NPA. If there is a restoring force proportional to distance and a minimum resident impending energy at steadiness, then a system displays a stable simple harmonic motion. Each small movement causes oscillations around equilibrium point when these requirements are met, making the motion predictable and stable. Regarding Eq. (10), surpassing parallel attitudes to those previously given^27–45^, the novel frequency might be attained as:

13 $$\Sigma^{2} (\Omega ) = \int\limits_{0}^{2\pi /\Sigma } {h(t)f_{3} (h,t)\,dt/\int\limits_{0}^{2\pi /\Sigma } {h^{2} (t)\,dt} }.$$

where

14 $$f_{3} (h,t) = \left( {\Omega^{2} - F\cos \sigma \,t} \right)h.$$

Utilizing MS, the novel whole frequency $\Sigma^{2}$ is approached and evaluated, as:

15 $$\Sigma^{2} (\sigma ) = \frac{{ - 4F + 4\Omega^{2} + \sigma^{2} + \sqrt {16F^{2} + 24F\sigma^{2} + \sigma^{4} - 32F\Omega^{2} - 8\sigma^{2} \Omega^{2} + 16\Omega^{4} } }}{8}.$$

### Validation of approach

When analyzing the DVMO, the goal of NPA is to transform the nonlinear ODE into a linear one devoid of the necessity of small approximations or adjustments. There are many features of NPA in the current oscillator such as:

1. It is simpler to accurately depict phenomena like parametric rolling, bifurcations, and sub-harmonic resonance when nonlinear restoring forces and damping are meticulously retained.
2. The equation of motion is numerically resolved for a given set of ICs. It has chaotic dynamics and huge rolling amplitudes that are outside the scope of perturbation methods.
3. This system can accommodate huge roll degrees, asymmetric hull shapes, and a range of complexity and it performs well under both periodic and irregular wave excitations.

The NS of the nonlinear DVMO as provided in Eq. (1) and NPA solution as provided in Eq. (6) are conveniently compared in Fig. 2 and Table 1. As seen , these figure and table show that the two solutions match well, with the highest error generated being 0.00463 when considering the subsequent data:

**Table 1:** Authorises the equivalent concerning the NS of $x(t)$ and its corresponding NPA one $u(t)$.

t	Nonlinear	Linear	Absolute error
0	0.1	0.1	0
5	0.00989381	0.00798517	0.00190864
10	-0.0342457	-0.0337403	0.00050538
15	-0.0145727	-0.0123268	0.00224585
20	0.00790585	0.00868507	0.000779216
25	0.0078219	0.00686845	0.000953449
30	-0.000452861	-0.00120018	0.000747315
35	-0.00302919	-0.00280741	0.000221785
40	-0.000789649	-0.00035479	0.000434858
45	0.000866148	0.000910646	0.0000444979
50	0.000564152	0.000382787	0.000181365

$$F = 0.05,\sigma = 2.5,G = 0.5,\mu_{1} = 0.1,\mu_{2} = 0.3,\alpha_{1} = 0.2,\,\alpha_{2} = 0.3,\alpha_{3} = 0.4,\,\,{\text{and}}\,\,A = 0.2.$$

As the accompanying graphic shows, the two curves have the best correlation when their forms closely match. This implies that transformations like translation, rotation, and scaling may be used to successfully superimpose one curve over the other. This connection may be understood using a number of concepts:

When the curvature, angle, and length distribution of two curves are similar along their trajectories, they form a strong relationship. As can be seen from their similarity, the curves have the same local properties, such as curvature radii and tangent directions. The matching may be objectively evaluated by comparing the two curves’ consistent locations. If these distances add up to as little as possible, the curves are said to be well-aligned. To assess this, methods such as the Hausdorff distance and point-to-point correspondence errors are frequently employed. The existence of an optimal conversion that aligns the two curves, including translation, rotation, and sometimes scaling, is indicated by an effective match. This change makes it possible to match any curve with its corresponding one, indicating a high degree of similarity. The curves’ corresponding structural features, such as periodicity, symmetry, or similar inflection points, are also necessary for the matching. Two curves will closely align if there is a consistent link between their attributes.

Table 1 demonstrates robust concordance between the numerical values obtained from both linear and nonlinear ODEs, indicating high precision and consistency across diverse computational methods or models. The robust correlation of results demonstrates that utilized numerical approaches are reliable and capable of handling a wide range of ODEs, regardless of their linearity. This strong agreement validates legality of the utilized approaches and confirms their effectiveness in practical situations.

### Stability analysis

Investigating the stability analysis of the given scenario as a result of NPA is our goal. For simplicity, comparison formulation’s stability graphs are shown using formula given in Eq. (12). This method works well, as seen in Fig. 2 and Table 1. Using the example discussed, it makes sense to examine replacement linear ODE as provided in Eq. (6) instead of nonlinear DVMO as produced in Eq. (1). Given that the current model can readily assess nonlinear stability, new technique is seen to be an intriguing, simple, and successful approach in this regard when compared to earlier methods. Numerous nonlinear formulae may be studied using this innovative method. The stability criterion can now be anticipated to resemble this:

16 $$\Sigma^{2} (\sigma ) > 0.$$

**Fig. 3:**
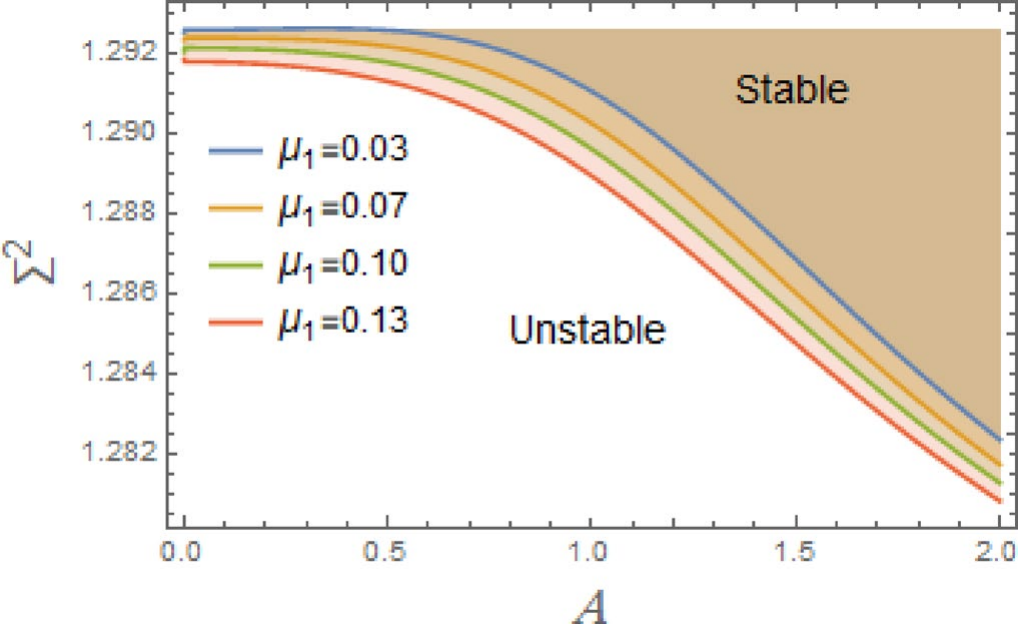
Displays the stabilizing effect of the linear damping coefficient $\mu_{1}$.

**Fig. 4:**
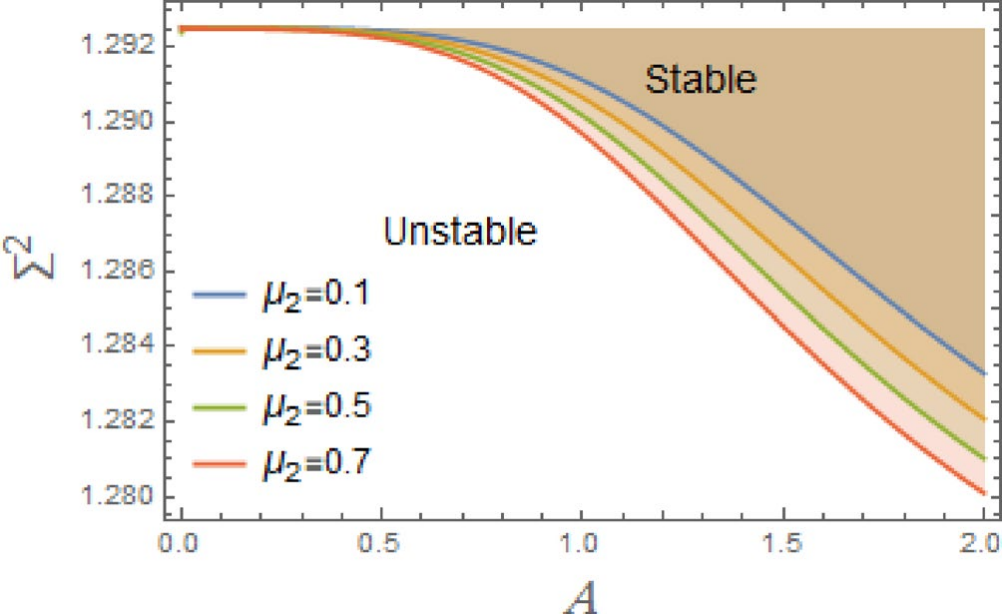
Displays the stabilizing effect of the nonlinear damping coefficient $\mu_{2}$.

**Fig. 5:**
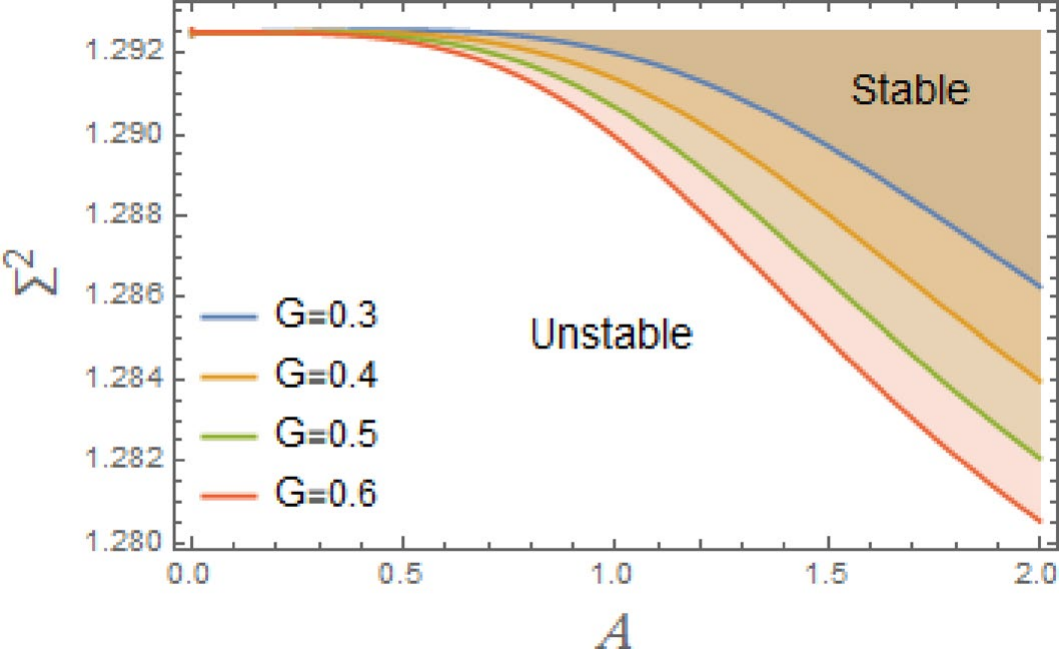
Displays the stabilizing effect of the gain coefficient $G$.

**Fig. 6:**
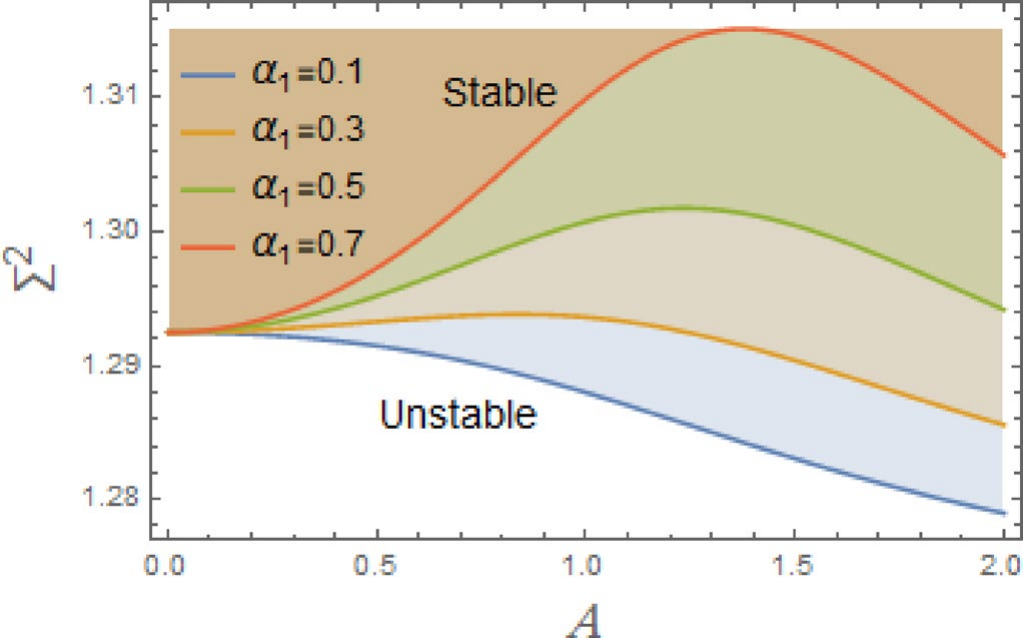
Displays the destabilizing effect of the nonlinear cubic coefficient $\alpha_{1}$.

**Fig. 7:**
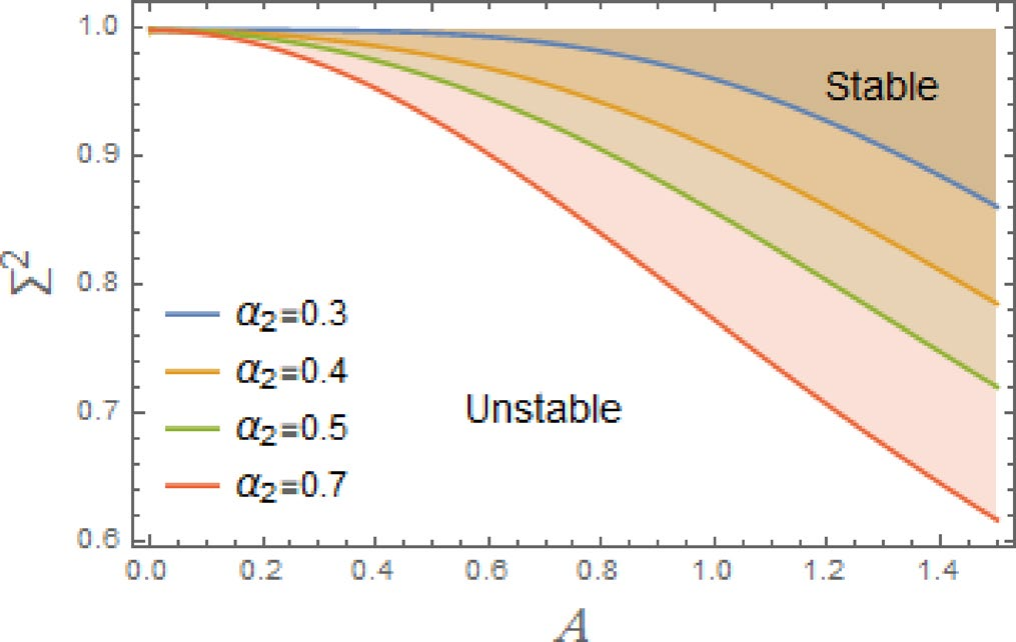
Displays the stabilizing effect of the nonlinear coefficient $\alpha_{2}$.

**Fig. 8:**
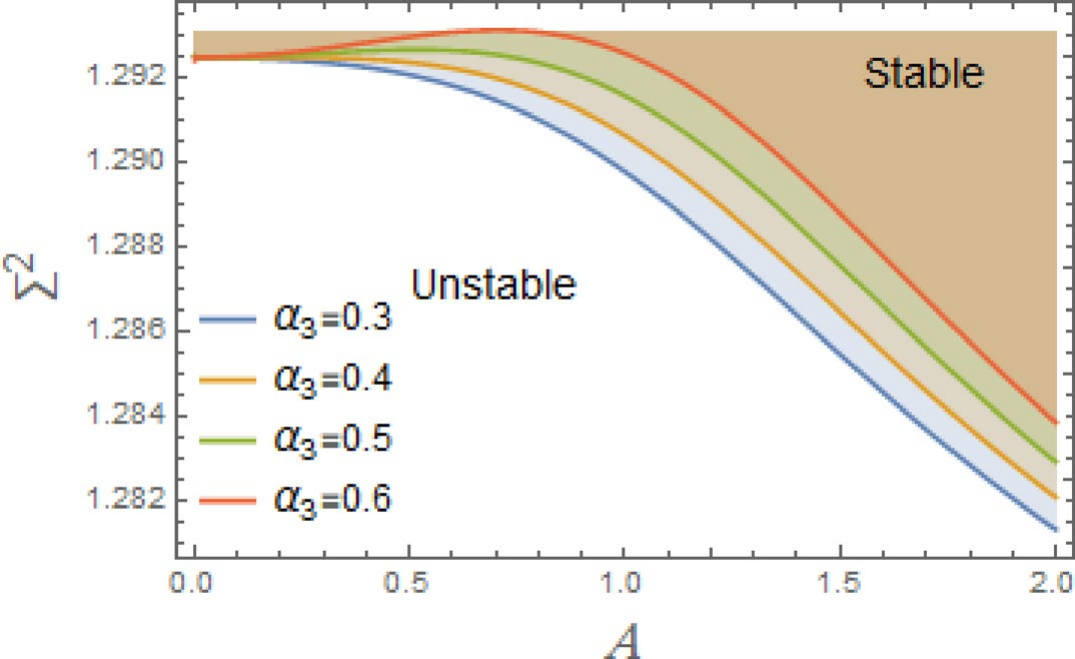
Displays the destabilizing effect of the nonlinear coefficient $\alpha_{3}$.

**Fig. 9:**
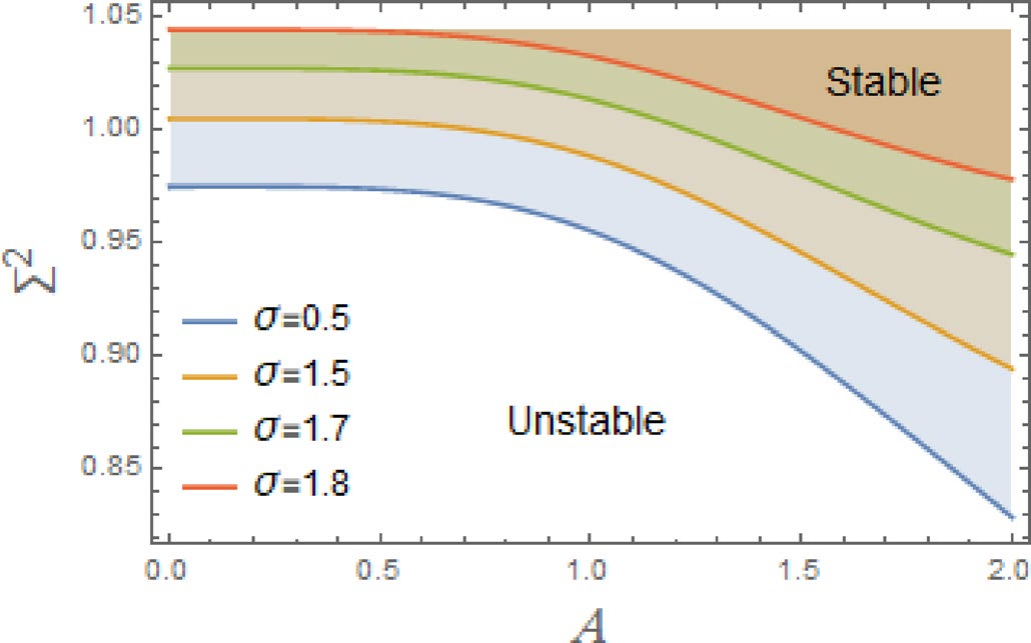
Displays the destabilizing effect of the excitation frequency $\sigma$.

**Fig. 10:**
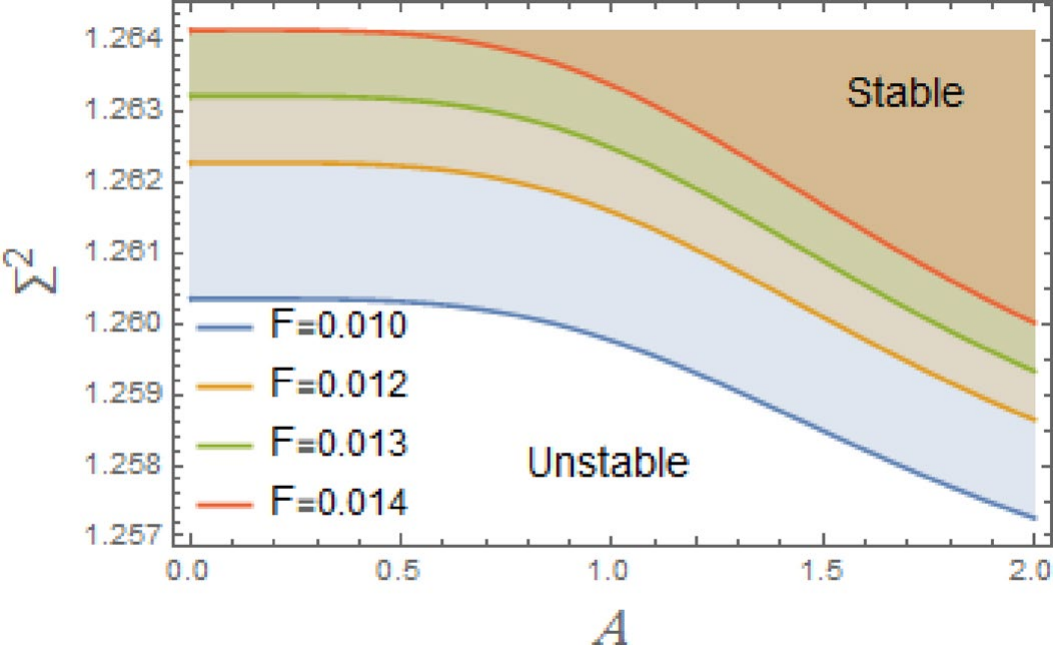
Displays the destabilizing effect of the excitation amplitude $F$.

In the subsequent, Figs. 3, 4, 5, 6, 7, 8, 9 and 10 are plotted to examine the stability graph in view of the constraint (16), where $\Sigma^{2} (\sigma )$ is signified alongside the preliminary amplitude $A$ in a minor interval from 0 to 2. As realized, the constraint $\Sigma^{2} (\sigma ) > 0$ seems as a transcendent function of parameters $F,\sigma ,G,\mu_{1} ,\mu_{2} ,$. $\alpha_{1} ,\,\alpha_{2} ,\alpha_{3} \,\,{\text{and}}\,\,A$ The efficacy of the MS is employed to authorize this circumstance by finding shape of stability by associating initial amplitude $A$ with drawing of $\Sigma^{2} (\sigma )$, as verified by these figures utilizing the following data:

$$F = 0.05,\sigma = 2.5,G = 0.5,\mu_{1} = 0.1,\mu_{2} = 0.3,\alpha_{1} = 0.2,\,\alpha_{2} = 0.3,\,\,\,{\text{and}}\,\,\alpha_{3} = 0.4,$$

The stable zones of a field with regard to the IC are better depicted by these graphs. The unstable segment is indicated by white region under curves, while stability zones are revealed by darker parts above them.

The restoring force, which continuously works to bring system back to its equilibrium position, is the source of stability in simple harmonic motion. According to Hooke’s Law, this force, which is frequently proportional to displacement, ensures that any departure from equilibrium results in an acceleration in its direction. In absence of other forces such as damping or driving factors, basic harmonic motion is essentially stable since system oscillates between maximum positive and negative displacements periodically and predictably. The system’s dynamic stability is demonstrated by the fact that it continues to oscillate in response to minor perturbations rather than deviating from equilibrium. However, by gradually reducing amplitude, practical factors like energy dissipation or roughness may affect the continuing stability and produce damped harmonic motion.

The impact of linear and nonlinear damping coefficients $\mu_{1}$,$\mu_{2}$, and $G$ on stability graph, according to condition (16), are shown in Figs. 3, 4 and 5. These graphs demonstrate that $\mu_{1}$,$\mu_{2}$, and $G$ have a stabilizing effect, expanding the stable zones as each of them grows. There are many factors affect steadying result of damping coefficients rising. To balance of energy produced by parametric excitation, damping term removes energy from system. By stabilizing system, this energy loss prevents oscillations from becoming excessively noisy. At resonant frequencies, damping reduces oscillations’ amplitude. In absence of damping, the system can expand endlessly in response to repeated inputs. Even little perturbations can cause chaotic behavior in nonlinear systems. Damping makes the behavior more predictable by reducing sensitivity to beginning circumstances, suppressing chaotic paths, and mitigating these disruptions. The stability zones inside the DVMO equation’s parameter space change when damping is introduced. In particular, it lessens the unstable areas, boosting the system’s stability over a larger range of parameters. Because it controls the oscillatory response and mitigates the effects of parametric stimulation, the linear damping coefficient is crucial for enhancing the stability of the nonlinear DVMO.

Figures 6, 7 and 8 are conspired to deliberate the impacts of nonlinear coefficients $$\alpha_{1} ,\,$$$$\alpha_{2}$$, and $$\alpha_{3}$$ on stability of system. The system is destabilized by nonlinear cubic coefficient $$\alpha_{1}$$, as detected in Fig. 6, in nonlinear DVMO, which alters parameters of stability and introduces possible chaotic dynamics and amplitude-dependent behavior. The third-order nonlinear coefficient has a significant effect on system’s dynamics and stability. The oscillator may become unstable in certain situations because of the amplitude-reliant rigidity, sub harmonic resonance, and potential for chaotic behavior. To lessen these disruptive impacts, operational and design methods must be effective. The growth of $$\alpha_{1}$$ causes the system to become nonlinearly stiff, introduces nonlinear resonance, jump events, and potential chaos, and can cause periodic forcing to destabilize equilibrium and sustained oscillations.

A stabilizing force is provided by the increasing of the nonlinear factor $\alpha_{2}$ in the nonlinear DVMO, as shown in Fig. 7. Increasing $\alpha_{2}$ has a stabilizing effect due to many reasons. The system gets more difficult to accelerate. This reduces abrupt motion shifts, particularly at large amplitudes, and causes a decrease in sensitivity to outside pressure. It aids in suppressing chaotic or resonant amplification by slowing down big oscillations, minimizing jump phenomena, and stabilizing limit cycles. Inertia, a self-regulating process, rises with displacement. When system begins to move too wildly, it becomes more “sluggish” to prevent uncontrolled oscillations. At higher amplitudes, effective natural frequency is lowered by additional inertia. This can shift system away from resonance by detuning it from the external stimulation frequency $\sigma$.

Furthermore, Fig. 8 indicates that nonlinear coefficient $\alpha_{3}$ decreases stability area which means that this parameter has a destabilizing effect on the discussed oscillator. This effect is due to the rise of $\alpha_{3}$ means that the phrase indicates that the restoring force depends on the square of the velocity in addition to the displacement. The restoring force intensifies with increasing velocity (because both contribute to this force). Complex behavior can result from this nonlinear relationship between displacement and velocity, particularly at high velocities. Small perturbations may be amplified by term, especially when velocity is high. In some situations, this can result in exponential growth and increase system’s sensitivity to beginning circumstances. Bifurcations or abrupt changes between several dynamical states, particularly close to resonance, can result from coupling of displacement and velocity. Chaos is strongly driven by combined effects of velocity dependence and nonlinear stiffness. It may result in a sensitive reliance on starting conditions, whereby little variations in starting conditions produce wildly disparate results. Under specific driving frequencies, this term is likely to cause chaotic attractors or nonlinear resonance in the system. Limit cycle bifurcations may occur in system when $\alpha_{3}$ rises, resulting in unstable oscillations at larger amplitudes. In response to slight variations in external stimuli, these unstable oscillations may transition between sub harmonic and super harmonic states, resulting in abrupt amplitude fluctuations.

The DVMO stability is significantly impacted by the excitation frequency $\sigma$. Figure 9 shows that the rise of $\sigma$ in a minor range from 0.5 to 1.8 causes a significant decay in the stability of the system. A parametric excitation is signified by the term $- xF\cos \sigma t$, where the forcing is proportional to the displacement, and the frequency $\sigma$ governs the oscillation of this forcing term. This effect is due to several interpretations.

(i) Resonance is most well-known destabilizing consequence of rising $\sigma$. The oscillation amplitude may increase uncontrolled when natural frequency of system matches exterior driving frequency $\sigma$. Resonance can get more complicated of a nonlinear system like DVMO, resulting in numerous resonance windows, sub harmonic resonance, or super harmonic resonance.
(ii) The system may undergo energy amplification as $\sigma$ gets closer to its native frequency (or its harmonics). If the driving force becomes resonant with response of the system, this may lead to oscillations of significant amplitude. This is particularly disruptive in nonlinear systems, where response may become chaotic or non-sinusoidal in addition to increasing in amplitude.
(iii) Bifurcations (qualitative changes in system dynamics) may arise when the driving frequency $\sigma$ increases. These might result in abrupt oscillation amplitude jumps, which could alter behavior on a broad scale (also known as jump resonance). Periodic solutions may become unstable or chaotic dynamics may result from the system’s ability to transition between states (for example, from low to high amplitude).
(iv) A change in $\sigma$ can result in a phase shift between the driving force and displacement in nonlinear systems. Higher values of $\sigma$ may result in chaotic motion when response of system shows unexpected oscillations and becomes extremely sensitive to beginning circumstances. This leads to uncontrollable behavior that lacks consistency and simple regularity.
(v) Complex resonance dynamics may result from nonlinear processes like amplitude-dependent frequency shifts as $\sigma$ rise. The system could react to several harmonic frequencies and show jump phenomena or sub harmonic oscillations. By forcing system into higher-order resonances, a rise in $\sigma$ can intensify the amplification of specific modes.

The stability zones decrease as excitation coefficient $F$ increases, as seen in Fig. 10. While other factors remain constant, stability regions dramatically drop as $F$ increases in a relatively narrow range, between 0.01 and 0.014. This influence demonstrates destabilizing effect of phenomenon. Excitation parameters have a destabilizing effect because they regulate parametric excitation’s amplitude. Increasingly $F$ makes system more susceptible to parametric resonance, which exacerbates oscillations in regions of Mathieu instability. Furthermore, these zones expand in parameter space with rising $F$, making the system more vulnerable to instability across a wider range of excitation frequencies. Larger excitation essentially makes instability worse, perhaps leading to massive oscillations, bifurcations, or chaotic behavior. The external force gets greater as $F$ rises, which may cause system to oscillate more. These greater oscillations can produce nonlinear responses in a nonlinear system in addition to being amplified linearly. When system is close to resonance, oscillations’ amplitude might increase more quickly. The system can reach a resonance with driving frequency $\sigma$ if $F$ is large enough. Jump events, in which system abruptly changes between various oscillation amplitudes or states, can result from resonance in nonlinear systems. A bifurcation may result from this, in which little variations in $F$ or $\sigma$ stiffness and damping factors, large $F$ can push the system into areas of unstable periodic solutions or even chaos. The system may be forced into areas where equilibrium points become unstable if $F$ is sufficiently large. Growing $F$ can make limit cycles unstable in the presence of significant nonlinearities or without damping, resulting in prolonged large-amplitude oscillations or even chaotic behavior. When driving force increases above a certain threshold, system may switch from steady oscillations to chaotic motion.

For more convenience, Table 2 shows that the nonlinear damping coefficient $G$ is optimally altered based on other system parameters like the excitation parameters $F$ and σ. From this table one can noted that the values of $G$ swings between increasing and decreasing with the growth of $\sigma$, and $F$. Furthermore in some ranges the value of $G$ stays constant.

**Table 2:** The values of the gain factor $G$ with the variation of $F$ and $\sigma$.

$F$	$\sigma$	$G$
0.05	0.5	1.36947*10^19
"	1	1.1984*10^18
"	1.5	2.60458*10^19
"	2	7.58171*10^17
"	2.5	6.89246*10^16
"	3	6.89246*10^16
"	3.5	6.26587*10^15
"	4	6.26587*10^15
"	4.5	6.26587*10^15
"	5	6.26587*10^15
0.01	2.5	6.26587*10^15
0.02	"	6.89246*10^16
0.03	"	6.89246*10^16
0.04	"	6.89246*10^16
0.05	"	6.89246*10^16
0.06	"	6.89246*10^16
0.07	"	4.13548*10^17
0.08	"	4.13548*10^17
0.09	"	4.13548*10^17
0.1	"	6.89246*10^16

### Time history examination

**Fig. 11:**
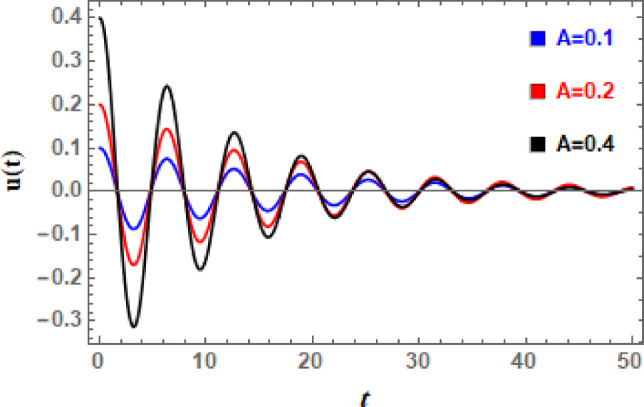
Displays the effect of *A* on the $u(t)$ performance.

**Fig. 12:**
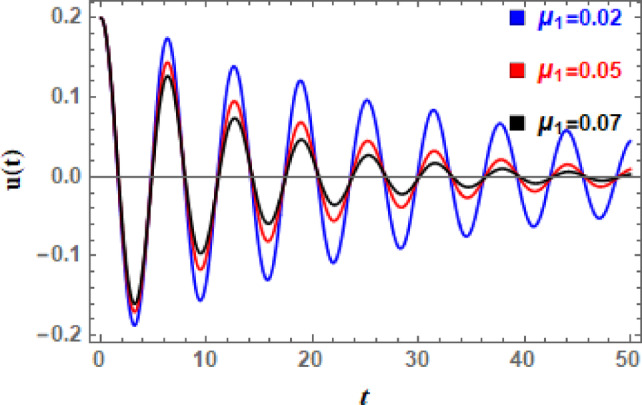
Shows the impact of $\mu_{1}$ on the $u(t)$ performance.

**Fig. 13:**
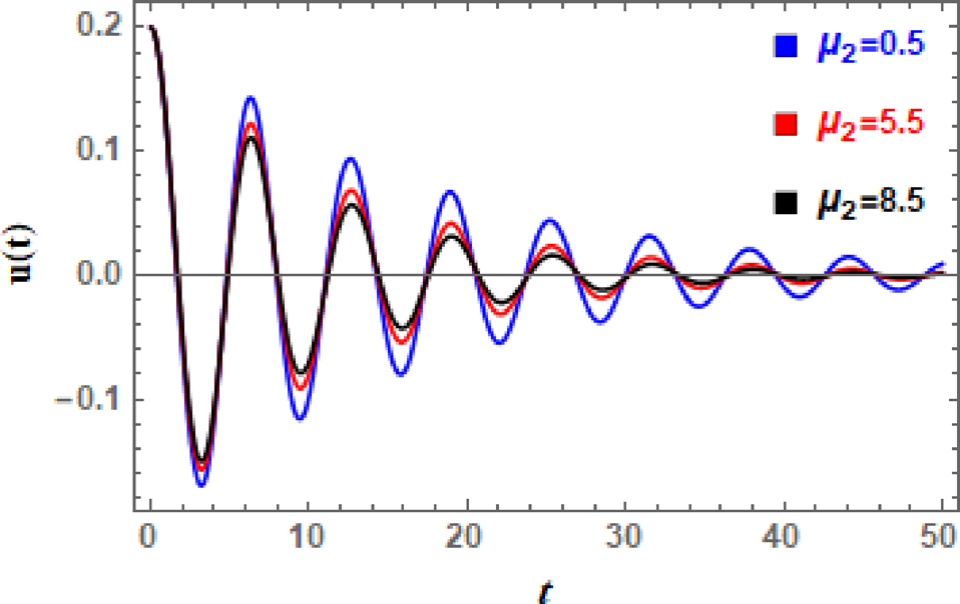
Illustrates the influence of $\mu_{2}$ on the $u(t)$ performance.

**Fig. 14:**
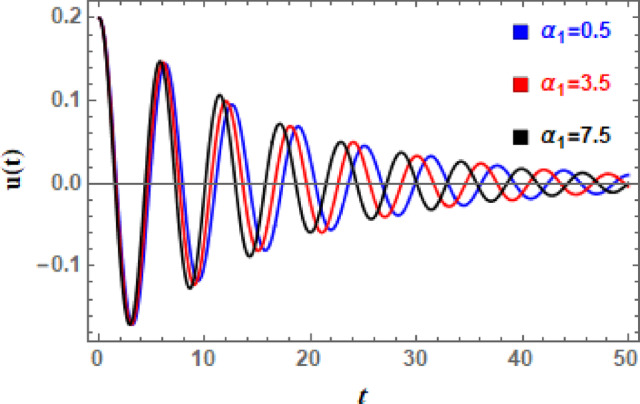
Displays the influence of $\alpha_{1}$ on the $u(t)$ performance.

**Fig. 15:**
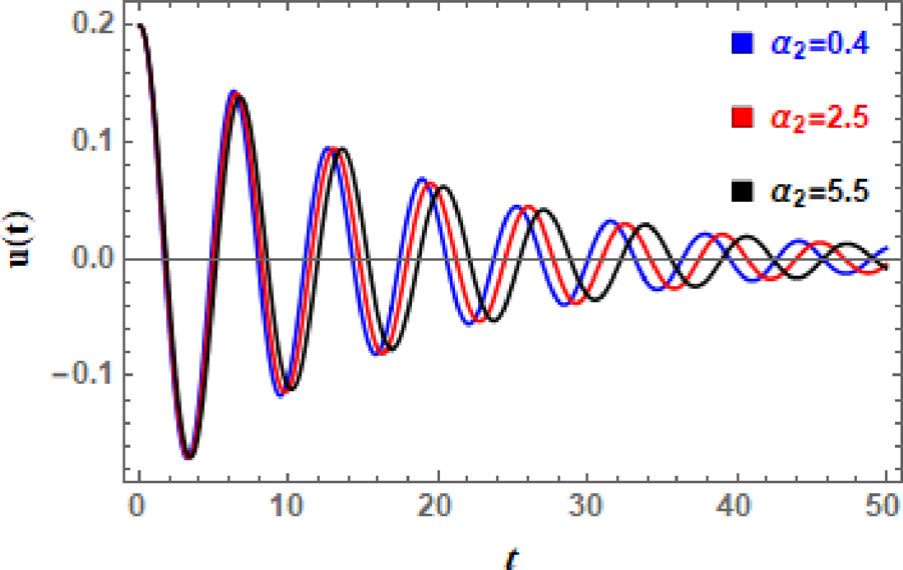
Illustrates the influence of mass-type coefficient $\alpha_{2}$ on the $u(t)$ performance.

**Fig. 16:**
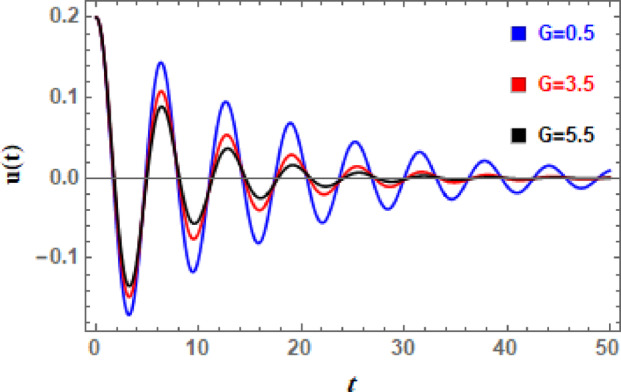
Illustrates the influence of the nonlinear cubic damping coefficient $G$ on the $u(t)$ performance.

In Figs. 11, 12, 13, 14, 15, and 16, the damped DVMO solutions as specified in Eq. (1) using NPA are visually represented vs. time (time historical graphs) using the following data:

$$F = 0.05,\sigma = 2.5,G = 0.5,\mu_{1} = 0.1,\mu_{2} = 0.3,\alpha_{1} = 0.2,\,\alpha_{2} = 0.3,\alpha_{3} = 0.4,\,\,{\text{and}}\,\,A = 0.2,$$

which are different depending on characteristic that is displayed. The temporal history of all these photographs shows that the wave amplitudes are steadily declining with time, and oscillations are becoming less pronounced. This is understandable due to damping force.

It is evident from Fig. 11 that as A increases, damped waves’ amplitudes increase. As A rises, neither its wavelengths nor its quantity of vibrations will change. The increase of A also keeps waves’ transmission regular. In a damped system, periodic solution’s amplitude determines oscillation’s starting energy. Higher initial amplitudes decay more slowly with damping because it must release more stored energy. Degradation rates may be changed by damping and large amplitudes because they cause oscillation frequency to diverge from natural frequency. In early time history oscillations are more noticeable at higher periodic solution amplitudes. Finally, oscillations settle into a periodic solution with reduced amplitude if force is applied. If it is unforced, they eventually disappear completely due to damping.

Furthermore, Fig. 12 indicates effect of linear damping element $\mu_{1}$ on the temporal dispersal of $u(t)$. This image shows that, as $\mu_{1}$ grows, the wave’s decaying rate increases as well, which makes sense. Furthermore, as grows, amplitudes augment while wavelengths and frequency of oscillations remain unchanged. When linear damping coefficient is increased, oscillations are quickly stopping. Motion becomes negligible; external pushing might not be sufficient to overcome damping. Additionally, the system may look over damped with only slight oscillations or a gradual decrease to equilibrium as $\mu_{1}$ increases. The current oscillator’s nonlinear damping accounts of velocity-dependent energy dissipation techniques, which become increasingly significant at higher speeds. It has a distinct influence from linear factors and plays a crucial role in figuring out oscillator dynamics, especially of large amplitude movements. Consequently, the influence of nonlinear damping factor $\mu_{2}$ is depicted in Fig. 13, from which it can be inferred that oscillations’ amplitudes are somewhat enhanced but their wavelengths and number stay unchanged. With an increase in $\mu_{2}$, waves decay more, although the ratio is smaller than when $\mu_{1}$ grows.

To further examine influence of nonlinear cubic factor $\alpha_{1}$ on DVMO’s temporal manner, Fig. 14 is shown. It can be seen from Fig. 14 that as cubic coefficient $\alpha_{1}$ develops, number of oscillations, wave amplitudes, and wavelengths are very constant. In the meantime, the expansion of $\alpha_{1}$ is observed to cause a minor degradation in the entire wave. The stiffness increases with displacement as $\alpha_{1}$ rises, resulting in a strong spring. The frequency-amplitude relationship is caused by natural frequency increasing with amplitude. Accordingly, time history can exhibit amplitude saturation or jump events, abrupt peaks or “flattened” troughs, asymmetric waveforms, and, under extreme forcing, bifurcations or chaos. Furthermore, Fig. 15 scrutinizes the influence of the nonlinear inertia or mass-type coefficient $\alpha_{2}$ on the time history of DVMO. From Fig. 15, it is detected that, as effect of $\alpha_{1}$, amplitudes, wavelengths, and number of oscillations, are nearly unvarying according to expansion of $\alpha_{2}$. But, contrary to effect of $\alpha_{1}$, a slight enhancement in total wave creates with development of $\alpha_{2}$. The mass increases with displacement as $\alpha_{2}$ rises because effective inertia rises with rise of the term $x^{2}$. This prevents fast acceleration by slowing down system’s reaction at bigger amplitudes. It adds a stabilizing effect, particularly when oscillations are significant. The system has a tendency to remain confined, and oscillations may become less intense. Increases in $\alpha_{2}$ can lessen the mayhem that other nonlinear terms cause.

Finally, influence of cubic damping factor $G$ on temporary configuration is indicated in Fig. 16. This effect is similar to the effects of $\mu_{1}$ and $\mu_{2}$ on the oscillations of $u(t)$. In current system, parameter $G$ functions as an energy sink that is reliant on velocity. This term makes a minimal contribution at low velocities. It helps regulate excessive oscillations or chaotic behavior because it aggressively opposes motion at high velocity. As $G$ increases, large-velocity motion is strongly suppressed, functioning as a velocity brake. Even with intense pushing, it may totally suppress chaotic or resonant oscillations. The system prefers constant motion with low amplitude and low velocity. If the oscillator is nonlinear, it may appear nearly over damped. Overall, the damping terms shape the time history in complementary but fundamentally distinct ways. An exponential decay (or decreased growth rate in the unstable parametric region) and a nearly constant phase lag are caused by the linear viscous term , which provides amplitude-independent, frequency-proportional energy loss. Increasing $\mu_{1}$ increases the effective damping ratio, pushes the system toward the stable side of the Mathieu-type boundary, and slows/suppresses resonance. The nonlinear quadratic and cubic damping, on the other hand, limits post-bifurcation growth, lowers peak amplitudes, and tends to smooth or clip transient peaks that would otherwise grow under parametric excitation. It is weak for small oscillations but becomes dominant at large amplitudes due to amplitude-dependent dissipation (energy loss scales strongly with velocity).

### Phase plane arrangement

**Fig. 17:**
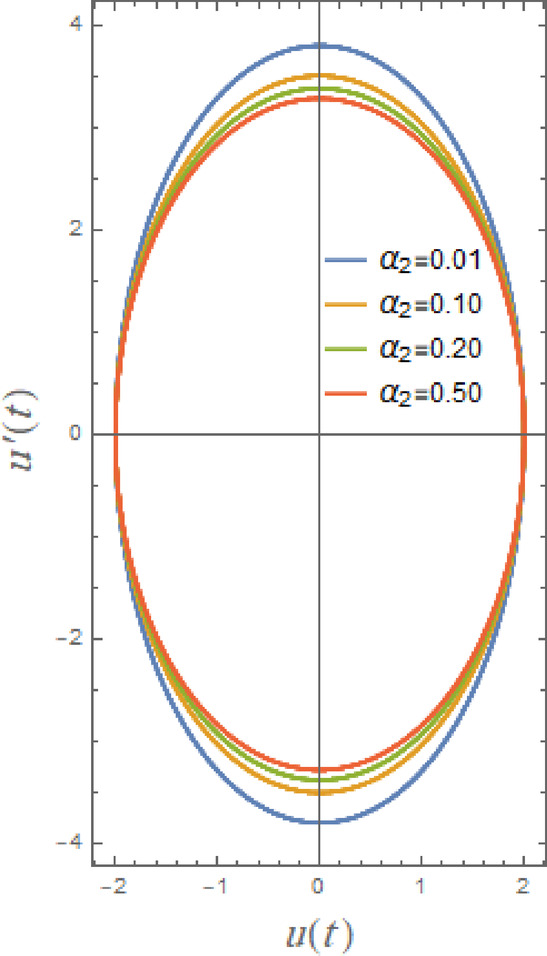
Displays the phase plane $uu^{\prime}$ under the influence of $\alpha_{2}$.

**Fig. 18:**
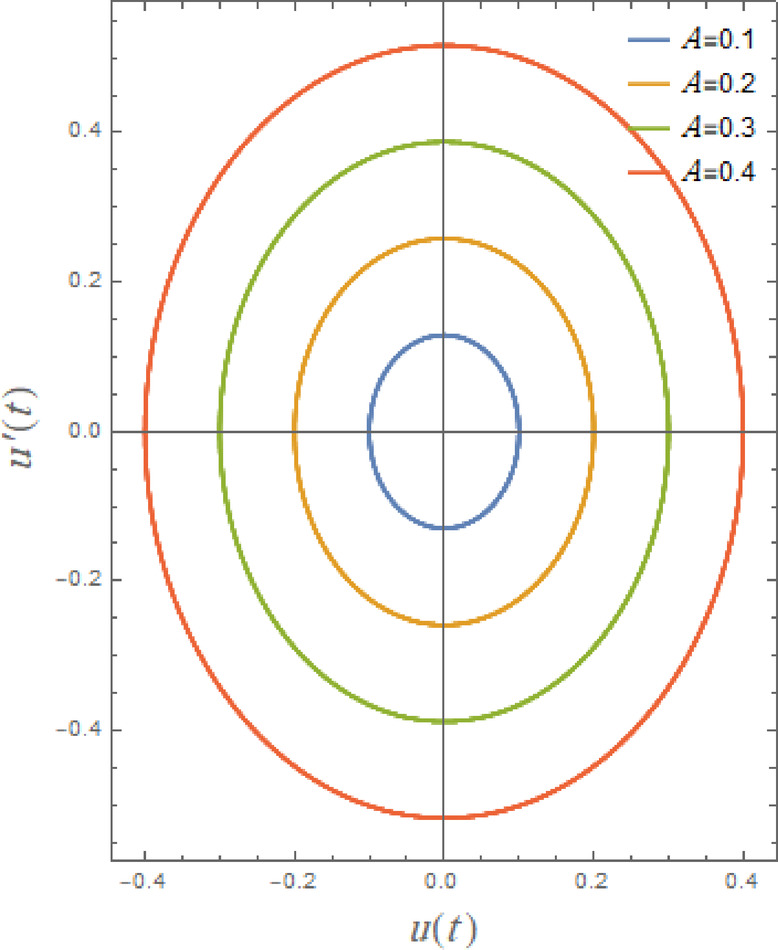
Illustrates the phase plane $uu^{\prime}$ under the influence of $A$.

**Fig. 19:**
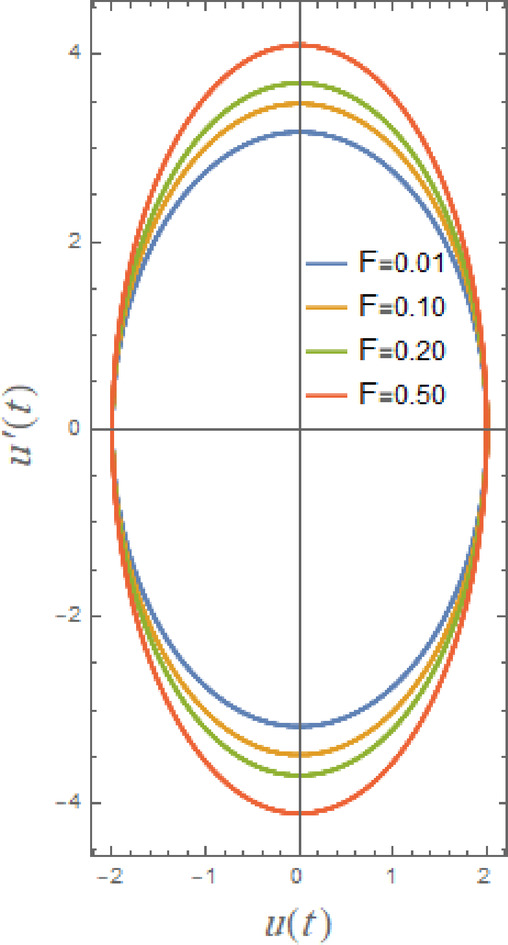
Displays the phase plane $uu^{\prime}$ under the influence of $F$.

**Fig. 20:**
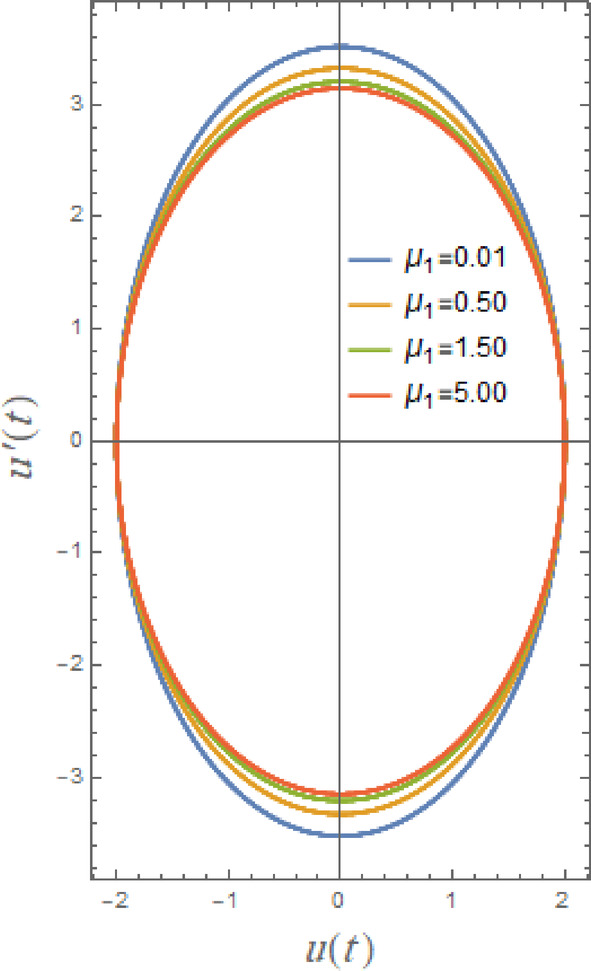
Shows the phase plane $uu^{\prime}$ under the influence of $\mu_{1}$.

**Fig. 21:**
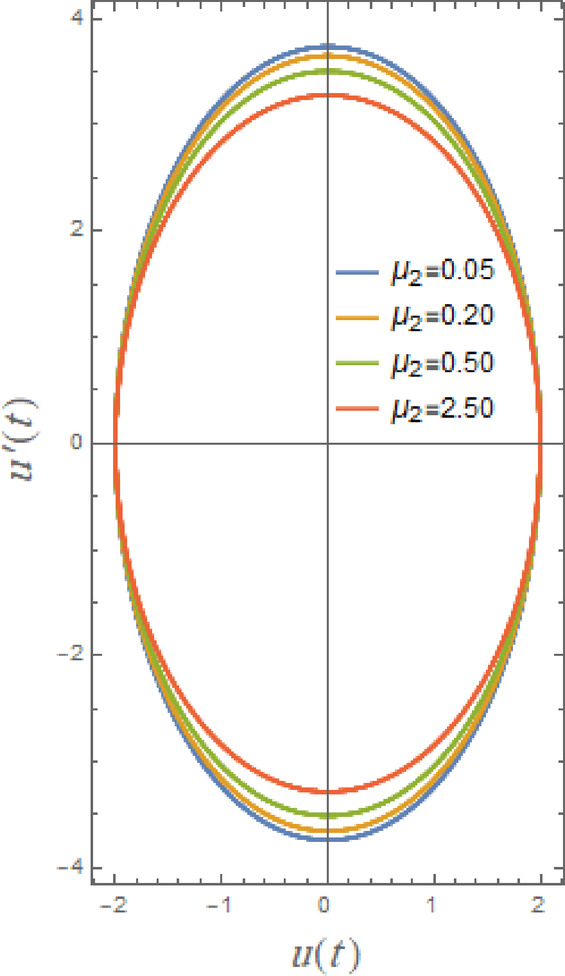
Shows the phase plane $uu^{\prime}$ under the influence of $\mu_{2}$.

**Fig. 22:**
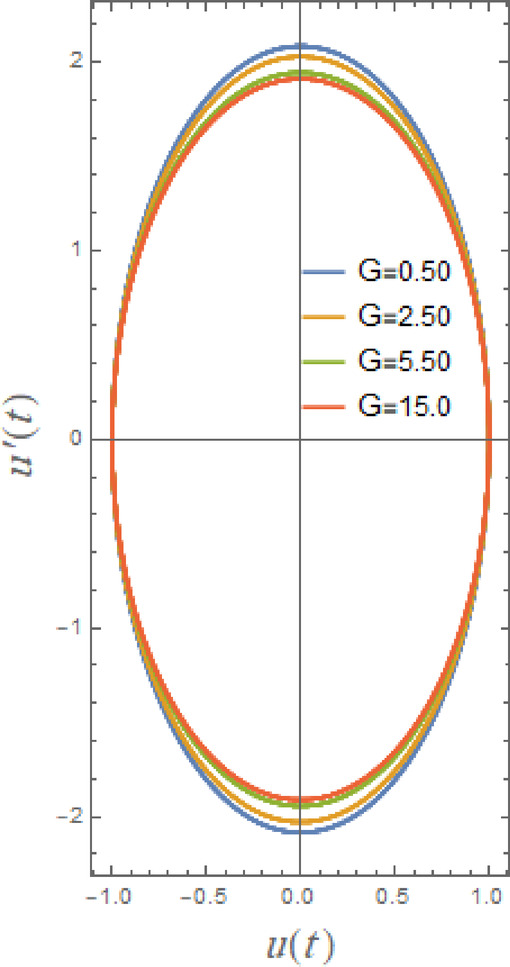
Displays the phase plane $uu^{\prime}$ under the influence of $G$.

In what follows, Figs. 17, 18, 19, 20, 21, and 22, demonstrate phase plane diagrams to show parametric relationships between equivalent function and its time derivative in plane in damped situation. Instead of charting location and velocity independently across time, it displays the system’s state in terms of both characteristics. This figure shows system’s current status at each point in time. The trajectory that is followed throughout time illustrates dynamic evolution of system.

The benefits in studying the phase plane diagrams may be summarized in:

1. **Stability**: Spiral inward means stable mode, meanwhile spiral outward means unstable mode.
2. **Periodic Motion**: Closed loops indicate limit cycles or steady oscillations.
3. **Chaos**: Extraordinary attractors or twisted paths.
4. **Damping**: Trajectories contract toward equilibrium.
5. **Nonlinearity**: Irregularity or distortion of typical circular/elliptical paths.

In Figs. 17, 18, 19, 20, 21 and 22, as different parameters increase, solutions’ stability or steady-state properties are displayed by symmetric circular loops around vertical or horizontal axes. Figure 17 here determines influence of nonlinear parameter $\alpha_{2}$ which enhances restoring force in DVMO as identified in Eq. (1). The shrinking of phase plane trajectories with increasing $\alpha_{2}$ is a feature of nonlinear inertial damping or mass stabilization. As $\alpha_{2}$ rises, system acquires resistance to motion with high displacement. High-amplitude motion is dampened as a result, lowering both *u* and *u*˙. A smaller loop or tighter spiral in the phase plane is the end outcome. The system acts as though it has nonlinear inertia brakes at high $\alpha_{2}$. Huge accelerations are resisted by system even when external pushing is applied, which speeds up energy loss from huge oscillations. This results in lesser displacement excursions, decreased velocity, and quicker settling.

The phase plane graphs $u(t)$ against $u^{\prime}(t)$ due to the impact of the initial amplitude $A$ as shown in Fig. 18. This graphic clearly shows that, as the trajectories ascend, bounded curves are greatly enlarging around center. More initial energy is received by system when starting amplitude is increased. More energy leads to larger motion, which causes system to begin farther out of equilibrium. It must move faster and farther in both directions. Consequently, more of the plane is covered by the phase trajectory as it expands outward.

The type and extent of trajectories of current solution are directly influenced by excitation magnitude. The influence depends on interplay between system’s damping, amplitude, natural frequency, and somewhat nonlinearity. Consequently, Fig. 19 illustrates how phase plane diagram is impacted by excitation amplitude coefficient $F$. The loops get smaller as $F$ increases in narrow range of 0.01 to 0.5, as observed in Fig. 19. Physically, bigger $F$ causes initial spirals to be larger of high damping, but system soon returns to a stability mode, meaning that trajectories get smaller. The system’s energy input is determined by excitation amplitude. As displacement increases, larger loops in phase plane and greater oscillation amplitudes are results of increasing excitation amplitudes, as shown in Fig. 19.

Figures 20, 21 and 22 here clarify the influence of damping parameters $\mu_{1}$,$\mu_{2}$ and $G$, respectively. These parameters characterize main decaying parameters of DVMO which enhances stability of system as seen before through the Figs. 3, 4 and 5. From these figures, one may observe that phase plane’s pathways are tight with development of $\mu_{1}$ from 0.01 to 5.0. As seen in Fig. 20, damping factor is logically thought of as a stable factor that provides regular elliptical paths. Damping causes system to lose energy, which eventually reduces oscillations. While smaller damping prolongs oscillations, larger damping accelerates energy loss. The impact of nonlinear damping coefficients $\mu_{2}$, and $G$ is shown to have same effect in Figs. 21 and 22, but there is minimal trajectory decay, which is consistent with earlier findings in stability and temporal drawings. The damping terms in the phase plane dissipate mechanical energy to regulate the orbits of the system in terms of shape and trajectory evolution. Trajectories spiral smoothly toward the origin due to linear viscous damping, creating uniformly diminishing loops that represent a uniform energy loss every cycle; the system reaches equilibrium more quickly when the spirals are tighter. Due to amplitude-dependent dissipation brought about by the nonlinear damping term, large trajectories contract quickly while small ones decay more slowly because the damping force increases with velocity magnitude. As a result, spirals become deformed and non-circular, with the outer loops collapsing before the inner ones. By compressing the phase trajectories toward a compact, virtually circular area around the equilibrium point, nonlinear damping specifically inhibits large-amplitude oscillations, while boosting either damping term generally decreases orbit size and stabilizes the system.

## Analysis of chaotic behavior

Analyzing bifurcation diagrams, phase portraits, and Poincaré maps are thought to be useful methods in comprehending how dynamical systems behave. In order to understand intricate dynamics of chaotic systems, this part is divided into an analysis of bifurcation diagrams, phase portraits, and Poincaré maps^51–55^. These analytical techniques provide profound insights into system’s evolution and transformative behavior. Specifically, bifurcation diagrams serve as powerful visual representations that track how a system’s qualitative characteristics metamorphose across different parameter ranges, revealing transitions between diverse dynamical states, including periodic, period-doubling, quasi-periodic, and chaotic regimes, and enabling researchers to precisely delineate regions of systemic stability and instability. The significance of examining these tools can be stated as follows:

Bifurcation diagrams:

- Stability boundaries: Reveal critical parameter values where beam’s behavior fundamentally changes.
- Response prediction: Show how vibration amplitudes vary with changing excitation parameters.
- Chaos identification: Highlight transitions from periodic to chaotic responses, which is vital for preventing unpredictable structural behavior.
- Design parameter selection: Guide engineers in selecting safe operational ranges that avoid dangerous resonance conditions

Phase portraits:

- Complete dynamic visualization: Capture full state-space behavior (position and velocity) of the beam.
- Attractor identification: Reveal stable motion patterns that system naturally gravitates toward.
- Energy distribution: Show how energy flows between potential and kinetic forms during vibration.
- Transient analysis: Allow examination of how the beam approaches its steady-state behavior.

Poincaré maps:

- Dimensional reduction: Simplify complex continuous dynamics into discrete representations
- Periodic orbit detection: Clearly identify different types of periodic responses
- Chaos quantification: Provide measurable evidence of chaotic behavior through fractal structures
- Subtle behavior detection: Reveal fine dynamical features that might be missed in time histories

These analysis tools are especially important because parametrically excited cantilever beams can exhibit highly sensitive dependence on initial conditions and parameter values, making traditional linear analysis insufficient for accurate prediction and design.

Dynamical system’s behavior when a parameter change is visually depicted using bifurcation curves. To provide a measure of chaos and stability, Poincaré maps are also examined. The combination of bifurcation curves and Poincaré map analysis aids in prediction and understanding of complex processes like stability alterations or chaos. In this regard, bifurcation figures of $x$ under impact of the damping coefficient $\mu_{1}$, frequency of force $\sigma$, amplitudes of excitations $F$, and Poincaré maps have been created to illustrate the different motions.

**Fig. 23:**
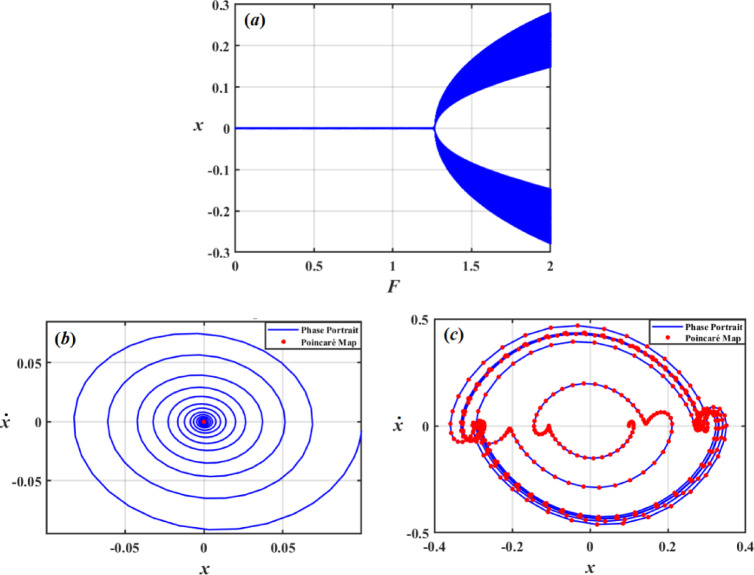
Shows: (*a*) bifurcation diagram for $x$ vs $F$, (*b*) Phase diagrams and Poincaré maps at $f = 0.05$, and (*c*) Phase diagrams and Poincaré maps at $f = 1.5$**.**

Figure 23 shows a simulated bifurcation diagram of the variable $x$. As $F$ grows, the system moves from a stable zone to chaotic performance, as seen in the bifurcation drawing in Fig. 23a. The system behaves periodically in first range, at $F \in [0,1.2]$. In second range at $F \in (1.2,2]$, the system is chaotic with extremely sensitive and erratic dynamics. The phase portraits and Poincaré maps of various values of $F$ are shown in Figs. 23b and c. The red dots in these plots indicate the Poincaré maps, which provide details about system’s stability and periodic behavior, while blue curves show system’s trajectory in phase space, reflecting phase portraits. A single red dot at centre of Fig. 23b represents a fixed point in Poincaré map, indicating that system is probably in a stable periodic or quasi-periodic state when $F = 0.05$. When $F = 1.5$, blue lines in Fig. 23c display a more intricate attractor with several red dots. This implies that the system is an attractor of chaos.

**Fig. 24:**
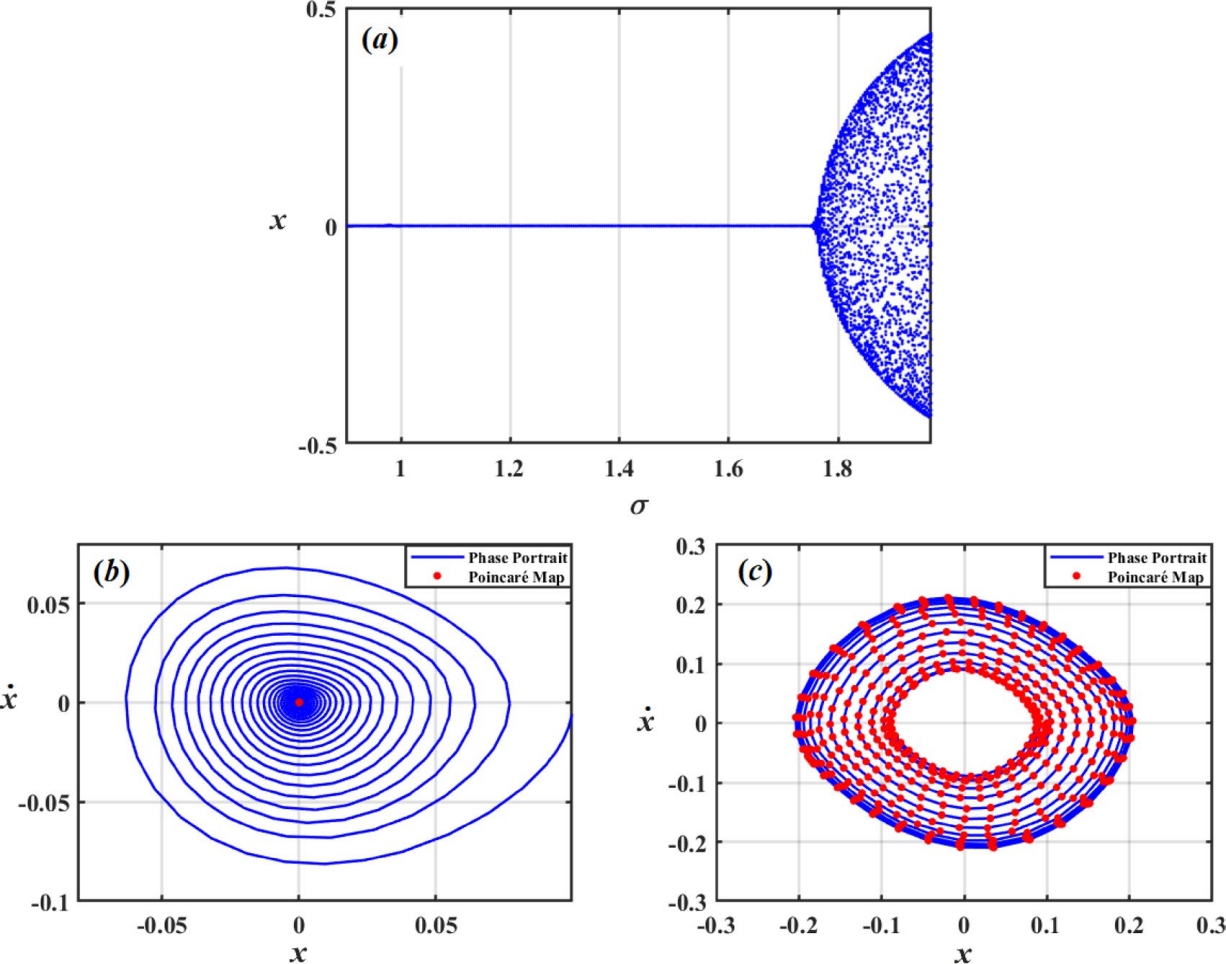
Displays (*a*) bifurcation diagram for $x$ vs $\sigma$, (*b*) Phase diagrams and Poincaré maps at $\sigma = 1$, and (*c*) Phase diagrams and Poincaré maps at $\sigma = 1.8$**.**

A bifurcation diagram illustrating evolution of variable $x$ with respect to force-frequency $\sigma$ is shown in Fig. 24a. It reveals a period of bifurcations at $\sigma < 1.7$ and beginning of chaos at $\sigma > 1.7$. The phase portrait in Fig. 24b suggests periodic or quasi-periodic behavior by displaying closed curves around equilibrium points that depict system’s attractor structure. Additionally, Fig. 24c displays a phase picture that shows beginning of chaos with a chaotic attractor in blue and strewn red Poincaré points occupying a larger area of phase space. These charts show how dynamics change from stable to chaotic as a function of $\sigma$.

**Fig. 25:**
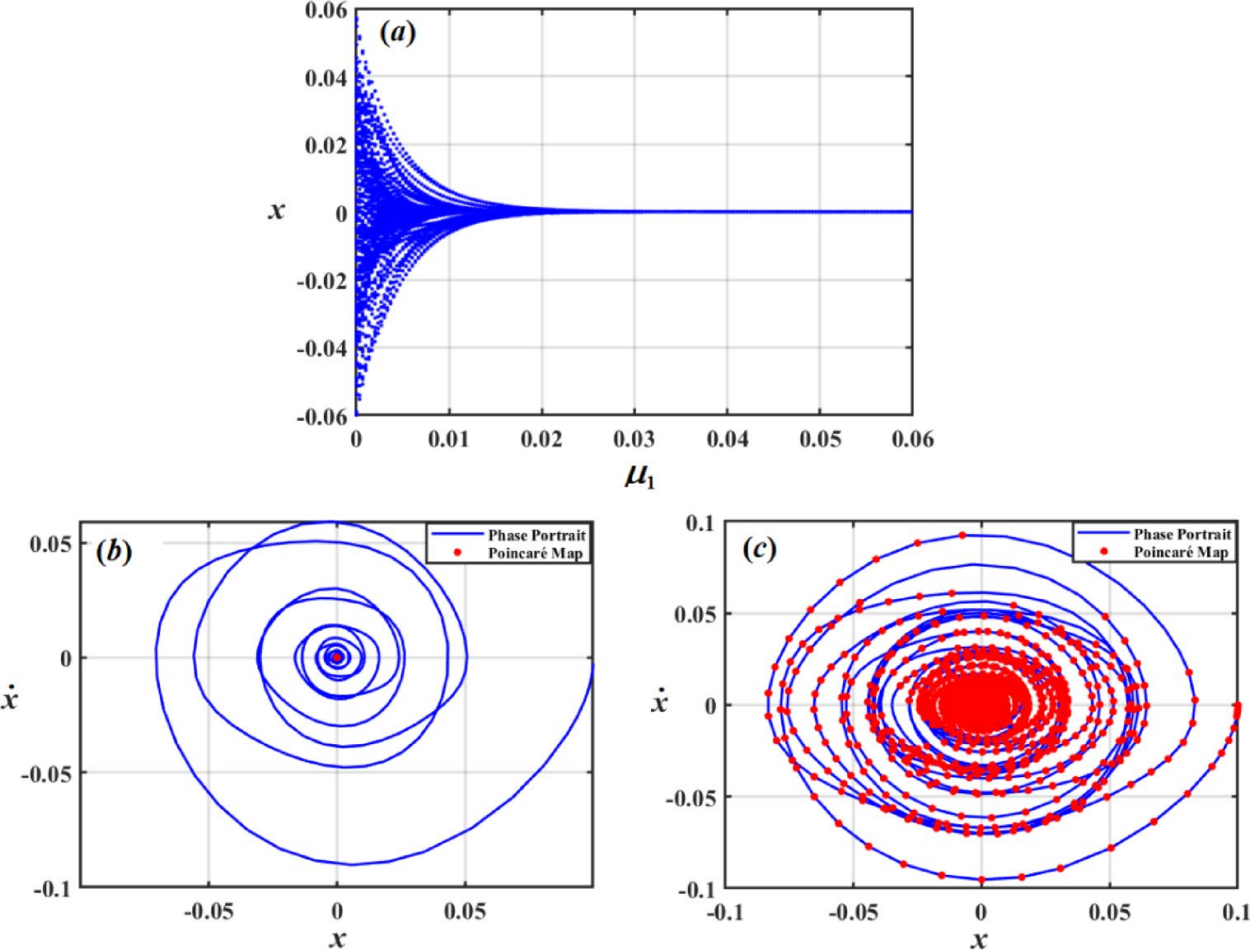
Demonstrates: (*a*) bifurcation diagram for $x$ vs $\mu_{1}$, (*b*) Phase diagrams and Poincaré maps at $\mu_{1} = 0.05$, and (*c*) Phase diagrams and Poincaré maps at $\mu_{1} = 0.01$.

A bifurcation diagram of variable $x$ vs. damping coefficient $\mu_{1}$ is displayed in Fig. 25a, where a rise in $\mu_{1}$ causes system to shift from chaotic behavior to a regular regime. The system displays chaotic behavior with high sensitivity and irregularity in the first range, at $\mu_{1} \in [0,0.025]$.The system enters a distinct periodic pattern in second range, at $\mu > 0.025$. The phase portrait is shown in Fig. 25b, which shows a closed-loop attractor with tightly spiralling trajectories around a central point, indicating a stable or quasi-periodic system. A red Poincaré section superimposed on the same phase space in Fig. 25c indicates the intersection of the trajectory with a chosen surface. Deterministic chaos is indicated by the irregular and dispersed dots.

## Conclusion

The examination of cantilever beam under parametric stimulation is crucial in engineering structures including bridges, aircraft wings, and MEMS, providing insights into vibration control, energy harvesting, and structural optimization. It has been shown that nonlinearities constrain the increase in responsiveness. The problem of reducing vibrations in a structure subjected to primary parametric excitation was examined. It was revealed that enhancement in sensitivity is constrained by nonlinearities. The study employed this knowledge to formulate a straightforward nonlinear feedback law aimed at mitigating vibrations of first mode of a cantilever beam subjected to primary parametric resonance. The core methodology is based on NPA, derived from HFF. This approach effectively transforms a weakly nonlinear oscillator described by a second-order nonlinear ODE into a linear one. The objective of this study was to move away from conventional perturbation techniques and provide unrestricted approximate solutions of small amplitude parametric components. To obtain successive approximations of solutions to parametric nonlinear fluctuations, it is crucial to swiftly evaluate frequency-amplitude function. The derived parametric equation demonstrates strong concordance with original equation when validated by MS. The stability behavior was examined in various settings. The current methodology diminished mathematical complexity, hence enhancing the execution of nonlinear parametric problems. Additionally, Poincaré map, phase portrait, and bifurcation were examined, collectively offering an extensive representation of system’s behavior across many phases. The subsequent elements were emphasized regarding unique methodology utilized or notable results attained:

(i) This study inspects cantilever beam subjected to parametric stimulation to develop a simple nonlinear feedback law experiencing primary parametric resonance.
(ii) A highly substantial accuracy was found and an extraordinary concordance with NS of original system was demonstrated using figures and tables when NPA was applied to convert present system’s nonlinear ODE into a linear one.
(iii) The system was found to be stabilized by increase of both linear and nonlinear damping coefficients when stability configuration was investigated under assessed stability necessity.
(iv) The system becomes less stable as excitation force’s parameters increase.
(v) The temporal behavior of models and advantageous outcomes of applied settings were main topics of debate. Additionally, elliptical trajectories that either diverge or converge around a single point were visible in phase plane curves.
(vi) Periodic behaviors were discovered when relevant parameters varied, which proved significant in a variety of applications.
(vii) There are many types of connected dynamical systems that can benefit from the current method, which is simple, appealing, promising, and powerful.

## Data Availability

All data generated or analysed during this study are included in this article.

## References

[1] Faraday, M. On a peculiar class of acoustical figures and on certain forms assumed by a group of particles upon vibrating elastic surfaces. *Philos. Trans. R. Soc. Lond.* **121**, 299–318 (1831).

[2] Mathieu, E. Mémoire Sur Le mouvement vibratoire d’une membrane de forme elliptique. *J. De Mathématiques Pures Et Appliquées*. **13**, 137–203 (1868).

[3] Nayfeh, A. H. & Mook, D. T. *Nonlinear Oscillations* (Wiley, 1979).

[4] Anderson, T. J., Nayfeh, A. H. & Balachandran, B. Experimental verification of the importance of the nonlinear curvature in the response of a cantilever beam. *J. Vib. Acoust.* **118**, 21–27 (1996).

[5] Miles, J. Parametric excitation of an internally resonant double pendulum. *J. Appl. Math. Phys.* **36**, 337–345 (1985).

[6] Nayfeh, A. H. & Jebril, A. E. S. The response of two degree-of-freedom systems with quadratic and cubic nonlinearities to multi-frequency parametric excitations. *J. Sound Vib.* **115**, 83–101 (1987).

[7] Jiang, T., Tang, Y., Xu, C. & Liu, W. A calibration and error evaluation method of a combined tracking-based vision measurement system for meter-scale components. *IEEE Trans. Industrial Inf. ***21**(6)*, PP* (99), 1–10 (2025).

[8] Borowska, M. et al. Microwave-assisted green synthesis of selenium nanoparticles using citrus extracts: insights into size-controlled formation and surface characteristics. *Colloids Surfaces A* **725** (1), 137516 (2025).

[9] Wang, G. et al. A wavelength-stabilized and quasi-common-path heterodyne grating interferometer with sub-nanometer precision. *IEEE TIM*. **73**, 1–9 (2024).

[10] Liu, Q., Duan, L., Ma, N. & Liu, G. Two-channel whispering gallery mode self-injection locking lasers for hybrid optically pumped atomic comagnetometers. *Measurement* **246**, 116674 (2025).

[11] Li, K. et al. Three-stage training strategy phase unwrapping method for high speckle noises. *Opt. Express*. **32** (27), 48895–48914 (2024). 39876182 10.1364/OE.544968

[12] Dong, Y. et al. High-speed PGC demodulation model and method with subnanometer displacement resolution in a fiber-optic micro-probe laser interferometer. *Photonics Res.* **12** (5), 921–931 (2024).

[13] Zhang, C. et al. Large-range displacement measurement in narrow space scenarios: fiber microprobe sensor with subnanometer accuracy. *Photonics Res.* **12** (9), 1877–1889 (2024).

[14] He, D., Xu, H., Wang, M. & Wang, T. Transmission and dissipation of vibration in a dynamic vibration absorber-roller system based on particle damping technology. *Chin. J. Mech. Eng.* **37** (1), 108 (2024).

[15] Yang, K., Li, X. & Zhang, Z. Ultrafast beam steering with microradian tracking accuracy via feed forward tuning and real-time error compensation. *Control Eng. Pract.* **165**, 106521 (2025).

[16] Cao, Y. et al. Multi-Functional Self-Sensing electronic gasket for structural health monitoring of transportation pipelines. *Adv. Funct. Mater.* **35** (20), 2412634 (2025).

[17] Huang, H., Huang, M., Zhang, W. & Yang, S. Experimental study of predamaged columns strengthened by HPFL and BSP under combined load cases. *Struct. Infrastruct. Eng.* **17** (9), 1210–1227 (2021).

[18] Yao, Y. et al. Seismic performance of steel-PEC spliced frame beam. *J. Constr. Steel Res.* **197**, 107456 (2022).

[19] Ghayesh, M. H., Kafiabad, H. A. & Reid, T. Sub- and super-critical nonlinear dynamics of a harmonically excited axially moving beam. *Int. J. Solids Struct.* **49** (1), 227–243 (2011).

[20] Kumar, P. A. & Dash, R. R. Nonlinear dynamics of a harmonically excited axially moving beam. *Int. J. Innovations Eng. Technol. (IJIET)*. **27** (2), 1–12 (2025).

[21] Ji, W. M., Wang, H. & Liu, M. Dynamics analysis of an impulsive stochastic model for Spruce budworm growth. *Appl. Math. Comput.* **19**, 336–359 (2021).

[22] He, J. H. Homotopy perturbation technique. *Comput. Methods Appl. Mech. Eng.* **178**, 257–262 (1999).

[23] Moatimid, G. M. & Amer, T. S. Analytical approximate solutions of a magnetic spherical pendulum: stability analysis. *J. Vib. Eng. Technol.* **11**, 2155–2165 (2023).

[24] He, J-H. The simplest approach to nonlinear oscillators. *Results Phys.* **15**, 102546 (2019).

[25] He, C-H. & Liu, C. A modified frequency-amplitude formulation for fractal vibration systems. *Fractals* **30** (03), 2250046 (2022).

[26] Niu, J-Y., Feng, G-Q. & Gepreel, K. A. A simple frequency formulation for fractal–fractional non-linear oscillators: a promising tool and its future challenge. *Front. Phys.* **11**, 1158121 (2023).

[27] Ismail, G. M., Moatimid, G. M. & Yamani, M. I. Periodic solutions of strongly nonlinear oscillators using he’s frequency formulation. *Eur. J. Pure Appl. Math.* **17** (3), 2154–2171 (2024).

[28] Moatimid, G. M. & Amer, T. S. Dynamical system of a time-delayed -Van der pole oscillator: a non-perturbative approach. *Sci. Rep.* **13**, 11942 (2023). 37488150 10.1038/s41598-023-38679-5 PMC10366103

[29] Moatimid, G. M., Amer, T. S. & Ellabban, Y. Y. A novel methodology for a time-delayed controller to prevent nonlinear system oscillations. *J. Low Freq. Noise Vib. Act. Control*. **43** (1), 525–542 (2024).

[30] Moatimid, G. M., Amer, T. S. & Galal, A. A. Studying highly nonlinear oscillators using the non-perturbative methodology. *Sci. Rep.* **13**, 20288 (2023). 37985730 10.1038/s41598-023-47519-5 PMC10662384

[31] Moatimid, G. M., El–Sayed, A. T. & Salman, H. F. Different controllers for suppressing oscillations of a hybrid oscillator via non–perturbative analysis. *Sci. Rep.* **14**, 307 (2024). 38172592 10.1038/s41598-023-50750-9 PMC10764832

[32] Alluhydan, K., Moatimid, G. M. & Amer, T. S. The non-perturbative approach in examining the motion of a simple pendulum associated with a rolling wheel with a time-delay. *Eur. J. Pure Appl. Math.* **17** (4), 3185–3208 (2024).

[33] Alluhydan, K., Moatimid, G. M., Amer, T. S. & Galal, A. A. A novel inspection of a time-delayed rolling of a rigid rod. *Eur. J. Pure Appl. Math.* **17** (4), 2878–2895 (2024).

[34] Alluhydan Kh, Moatimid, G. M., Amer, T. S. & Galal, A. A. Inspection of a time-delayed excited damping duffing oscillator. *Axioms* **13** (6), 416 (2024).

[35] Moatimid, G. M., Mohamed, M. A. A. & Elagamy, Kh. An innovative approach in inspecting a damped Mathieu cubic–quintic duffing oscillator. *J. Vib. Eng. Technol.* **12** (Suppl 2), S1831–S1848 (2024).

[36] Moatimid, G. M., Amer, T. S. & Galal, A. A. Inspection of some extremely nonlinear oscillators using an inventive approach. *J. Vib. Eng. Technol.* **12** (Suppl 2), S1211–S1221 (2024).

[37] Moatimid, G. M., Mohamed, M. A. A. & Elagamy, Kh. Insightful examination of some nonlinear classification linked with Mathieu oscillators. *J. Vib. Eng. Technol.* **13**, 173 (2025).

[38] Alanazy, A., Moatimid, G. M., Amer, T. S., Mohamed, M. A. A. & Abohamer, M. K. A novel procedure in scrutinizing a cantilever beam with tip mass: analytic and bifurcation. *Axioms* **14**, 16 (2025).

[39] Ismail, G. M., Moatimid, G. M., Alraddadi, I. & Kontomaris, S. V. Scrutinizing highly nonlinear oscillators using he’s frequency formula. *Sound Vib.* **59** (2), 2358 (2025).

[40] Alanazy, A., Moatimid, G. M. & Mohamed, M. A. A. Innovative methodology in scrutinizing nonlinear rolling ship in longitudinal waves. *Ocean Eng.* **327**, 120924 (2025).

[41] Moatimid, G. M., El-Bassiouny, A. F. & Mohamed, M. A. A. Novel approach in inspecting nonlinear rolling ship in longitudinal waves. *Ocean Eng.* **327**, 120975 (2025).

[42] Alanazy, A., Moatimid, G. M. & Mohamed, M. A. A. Insights in inspecting two-degree-of-freedom of Mathieu-cubic-quintic Duffing oscillator. *Eur. J. Pure Appl. Math.* **18** (2), 5662 (2025).

[43] Ismail, G. M., Moatimid, G. M., Kontomaris, S. V. & Cveticanin, L. A novel methodology for scrutinizing periodic solutions of some physical highly nonlinear oscillators. *Computation* **13**, 105 (2025).

[44] Moatimid, G. M., Amer, T. S. & Galal, A. A. An innovative methodology in analyzing certain pendulum oscillators. *Sci. Rep.* **15**, 15883 (2025). 40335614 10.1038/s41598-025-99645-x PMC12059020

[45] Alanazy, A., Moatimid, G. M., Amer, T. S. & Galal, A. A. Non-perturbative approach in scrutinizing nonlinear time-delay of Van der Pol-Duffing oscillator. *Eur. J. Pure Appl. Math.* **18** (1), 5495 (2025).

[46] Oueini, S. S. & Nayfeh, A. H. Single-mode control of a cantilever beam under principle excitation. *J. Sound Vib.* **224** (1), 33–47 (1999).

[47] Zavodeny, L. D. & Nayfeh, A. H. The nonlinear response of a slender beam carrying a lumped mass to a principal parametric excitation: theory and experiment. *Int. J. Non-Linear Mech.* **24**, 105–125 (1989).

[48] Elìas-Zúñiga, A. Exact solution of the cubic-quintic duffing oscillator. *Appl. Math. Model.* **37**, 2574–2579 (2013).

[49] He, J-H. Some asymptotic methods for strongly nonlinear equations. *Int. J. Mod. Phys. B*. **20**, 1141–1199 (2006).

[50] Geng, L. & Cai, X. C. He’s frequency formulation for nonlinear oscillators. *Eur. J. Phys.* **28**, 923–931 (2007).

[51] Abohamer, M. K., Awrejcewicz, J. & Amer, T. S. Modeling and analysis of a piezoelectric transducer embedded in a nonlinear damped dynamical system. *Nonlinear Dyn.* **111**, 8217–8234 (2023).

[52] Bahnasy, T. A. et al. Stability and bifurcation analysis of a 2DOF dynamical system with piezoelectric device and feedback control. *Sci. Rep.* **14**, 26477 (2024). 39488558 10.1038/s41598-024-75342-z PMC11531538

[53] Abohamer, M. K., Amer, T. S., Arab, A. & Galal, A. A. Analyzing the chaotic and stability behavior of a duffing oscillator excited by a sinusoidal external force. *J. Low Freq. Noise Vib. Act. Control*. **44** (2), 969–986 (2025).

[54] Amer, T. S., Abdelhfeez, S. A., Elbaz Rewan, F. & Abohamer, M. K. Exploring dynamics, stability, and bifurcation in a coupled damped oscillator with piezoelectric energy harvester. *J. Low Freq. Noise Vib. Act. Control*. **44** (2), 938–958 (2025).

[55] Abohamer, M. K. et al. On chaotic behavior, stability analysis, and vibration control of the Van der Pol–Mathieu–Duffing oscillator under parametric force and resonance. *J. Low Freq. Noise Vibr. Active Control*. 10.1177/14613484251341933 (2025).

